# Recent strategies for nano-based PTT combined with immunotherapy: from a biomaterial point of view

**DOI:** 10.7150/thno.56482

**Published:** 2021-06-04

**Authors:** Xingyue Huang, Yang Lu, Mingxue Guo, Shouying Du, Ning Han

**Affiliations:** School of Chinese Materia Medica, Beijing University of Chinese Medicine, Beijing 102488, China.

**Keywords:** photothermal therapy, immunotherapy, combination therapy, nanoplatforms, cancer

## Abstract

Cancer has been a great threat to humans for decades. Due to the limitations of monotherapy, combinational therapies such as photothermal therapy (PTT) and immunotherapy have gained increasing attention with expectation to overcome the shortfalls of each other and obtain satisfactory therapeutic outcomes. PTT can inhibit primary tumors by thermal ablation but usually fails to achieve complete eradication and cannot prevent metastasis and recurrence. Meanwhile, the efficacy of immunotherapy is usually attenuated by the weak immunogenicity of tumor and the immunosuppressive tumor microenvironment (ITM). Therefore, many recent studies have attempted to synergize PTT with immunotherapy in order to enhance the therapeutic efficacy. In this review, we aim to summarize the cutting-edge strategies in combining nano-based PTT with immunotherapy for cancer treatment. Herein, the combination strategies were mainly classified into four categories, including 1) nano-based PTT combined with antigens to induce host immune responses; 2) nano-based PTT in combination with immune adjuvants acting as *in situ* vaccines; 3) nano-based PTT synergized with immune checkpoint blockade or other regulators to relieve the ITM; 4) nano-based PTT combined with CAR-T therapy or cytokine therapy for tumor treatment. The characteristics of various photothermal agents and nanoplatforms as well as the immunological mechanisms for the synergism were also introduced in detail. Finally, we discussed the existing challenges and future prospects in combined PTT and immunotherapy.

## Introduction

Due to aging and population growth, there will be a significant rise in the annual number of new cancer cases from 18.1 million in 2018 to 29.4 million in 2040 1. Cancer has become a tremendous threat to people worldwide. Currently, the conventional strategies for tumor treatment mainly include surgery, radiotherapy and chemotherapy. All of them showed positive therapeutic effects on early tumors but had limited efficacy for advanced tumors with metastasis 2. Moreover, various side effects accompanied by these therapies also bring great challenges 3. Therefore, more effective treatment strategies with high specificity continue to be explored.

Photo-therapy, which harnesses the absorption of near-infrared (NIR) light (650-1350 nm) by photo-agents to induce a therapeutic response has drawn great attention. Upon NIR light irradiation, the hyperthermia generated by photothermal agents is the basis for photothermal therapy (PTT), while the reactive oxygen species (ROS) released by photo-sensitizers is the main mechanism for photodynamic therapy (PDT) 4. In this review, we mainly focused on recent advances in nano-based PTT combined with immunotherapy. The combination of PDT with immunotherapy was not included this time, but it has been introduced in other articles in details 5-8. PTT can reduce tumor burden through thermal ablation, and it has become an attractive option in cancer treatment 9. By locally exerting NIR light irradiation, the hyperthermia (>42 °C) generated by photothermal agents can cause apoptosis of cancer cells by destroying the cell membrane, damaging the cytoskeleton and inhibiting DNA synthesis 10-12. In addition, PTT was able to trigger host immunity, revealing promising prospects in combination with immunotherapy 13, 14. Moreover, tumors are more sensitive to heat than normal tissues due to oxygen deficit and the mild acidic microenvironment; thereby, this strengthens the selectivity of PTT to tumors 15. Research interests have been extensively concentrated on its applications and combination with other therapies, since PTT has advantages including high selectivity, non-resistance and minimal invasiveness 16, 17.

The key to achieve photo-ablation of tumor relies on the design of photothermal agents which should have strong NIR light absorbance, high photothermal conversion efficiency, good biocompatibility and sufficient accumulation within tumors. Currently, various types of inorganic and organic photothermal agents have been exploited for their ability to perform PTT on tumors. Inorganic photothermal agents mainly include gold nanomaterials, like gold nanoparticles (GNPs), gold nanorods (GNRs) and gold nanostars (AuNSs) 18, 19; carbon based materials, such as single-walled carbon nanotubes (SWCNTs), graphene and carbon dots (CD) 20, 21; sulfide metallic nanoparticles, for example, CuS and MnS_2_ 22; black phosphorus and etc. 23. Generally, they are characterized by high photothermal conversion efficiency and photothermal stability. Their photothermal property can be adjusted by varying their size, shape or doping with other elements 24. However, the slow degradation rate *in vivo* might cause potential toxicity 18. While the organic photothermal agents typically include small molecular dyes, such as indocyanine green (ICG) and IR780; polydopamine (pD), polyaniline (PANI) and polypyrrole nanoparticles 25. Those organic photothermal agents are usually degradable and have high biocompatibility; but some of them are still facing drawback of photobleaching. Moreover, photothermal agents are usually designed as nanoplatforms. Due to the nanoscale sizes or surface modification of targeting ligands such as antibodies, folic acid, peptides and hyaluronic acid 26-28), these photothermal agents could achieve passive or active targeted delivery to tumors, thereby enhancing the accumulation in tumors. Moreover, they can meanwhile serve as nanocarriers to load drugs, antigens or adjuvants, exhibiting potential for combinational therapy with other treatment modalities 29, 30.

Even though PTT could debulk the tumor volume rapidly, it is generally difficult to completely eradicate tumors with PTT alone for some reasons as follows: 1) The penetration depth for NIR light is limited. Typically, the penetration depth of an NIR laser of 808 nm was reported to be within several millimeters (mm) (normally less than 5 mm 31), which is difficult to reach the very inside of a large tumor. 2) Photobleaching after a short time period of laser irradiation leads to a reduction in photothermal efficacy, especially for organic small molecular dyes. 3) Long-term tumor remission was insufficient, and there are high risks of tumor relapse and metastasis. Therefore, combining PTT with other therapies was expected to overcome the above challenges.

The ability to evade immune system surveillance and passivate immunogenicity is the primary reason for the occurrence and development of tumors 32. Generally, there are three important phases in cancer immune surveillance: elimination, equilibrium and escape 33, 34. In the process of elimination, firstly, acute inflammatory responses triggered by tumor-associated antigens (TAAs) can promote the secretion of cytokines such as interleukin-12 (IL-12) and interferon-γ (IFN-γ), and induce the activation of dendritic cells (DCs). Then upon activation, DCs will migrate to the nearby lymph nodes (LNs), where they present tumor antigens and activate tumor-specific CD8^+^ cytotoxic T lymphocyte (CTLs) to kill tumor cells. During the phase of equilibrium, a long-lasting campaign between the immune system and cancer cells is established. Tumor cells with high immunogenicity are eradicated by the immune system, while others that can lower their immunogenicity by immune editing will survive. Consequently, immune escape occurred. Additionally, certain negative regulators, including the PD-L1 on tumor cells, interleukin 10 (IL-10), transforming growth factor β (TGF-β), regulatory T cells (Tregs) and myeloid-derived suppressor cells (MDSCs) in the tumor microenvironment (TME) can prevent the activation of immune cells and prevent the tumor infiltration of CTLs and antigen-presenting cells (APCs) 35, 36.

Recently, immunotherapy in which the body immune system is trained to recognize and fight against tumors has shown great potential for cancer treatment 37, especially for aggressive and metastatic tumors. Cancer immunotherapy relies on the efficient presentation of tumor antigens to T-cells to elicit a potent anti-tumor immune response and generate long-term immune memory, thereby inducing the killing of cancer cells and preventing cancer recurrence 38. Currently, cancer immunotherapy mainly includes the application of tumor vaccines, immune checkpoint blockade (ICB) and chimeric antigen receptor T cell (CAR-T) therapy, which can restrain the growth and metastasis of tumors either by strengthening the immune response or reversing the immunosuppressive microenvironment (ITM). However, despite the advantages of immunotherapy, it also has limitations. 1) Single immunotherapy is not effective for all types of cancer, and the therapeutic responses may vary between different patients. 2) The efficacy of immunotherapy for large tumors is generally limited due to the ITM, loss of immunogenicity for cancer cells and excessive tumor burden 39, 40. 3) ICB therapies only perform their therapeutic function on their associated pathways instead of priming the immune system to specific response 41. 4) The activation of anti-tumor responses after vaccination is low due to variations in antigen specificity between different tumors and patients 42. All of the above factors have led to the attenuated efficacy of single immunotherapy in the treatment of cancers.

On account of the characteristics of immunotherapy and PTT, a combination of these two therapies has been proposed for the following reasons: 1) Nano-based PTT is able to eliminate the primary tumor by thermal ablation and rapidly reduce the tumor burden. In addition, the hyperthermia generated by PTT can induce immunogenic cell death (ICD) and release damage-associated molecular patterns (DAMPs) or TAAs, thereby increasing the immunogenicity of residual tumor tissues to trigger immune responses. Moreover, these nanoplatforms can be easily taken up by DCs *via* endocytosis and promote the cross-presentation of antigens, which makes them promising vehicles for cancer immunotherapy 43. 2) Immunotherapy mainly involves the subsequent recruitment and maturation of DCs, promoting the secretion of pro-inflammatory cytokines and stimulating the CTLs to clear up residual tumor cells as well as establishing long-term immune memories to prevent metastasis and tumor recurrence. Typically, the strategies for combining nano-based PTT with immunotherapy mainly include 1) nano-based PTT combined with antigens to elicit immune responses; 2) nano-based PTT in combination with immune adjuvants acting as *in situ* vaccines; 3) nano-based PTT synergize with ICB or other regulators to relieve ITM. 4) nano-based PTT combined with other immunotherapies such as CAR-T therapy or cytokine therapy for cancer treatment. This article mainly reviewed the recent strategies of nanoplatform-based PTT combined with immunotherapies (Table 1) and discussed challenges and future prospects.

## Nano-based PTT combined with antigens to induce host immune responses

**Figure 1:**
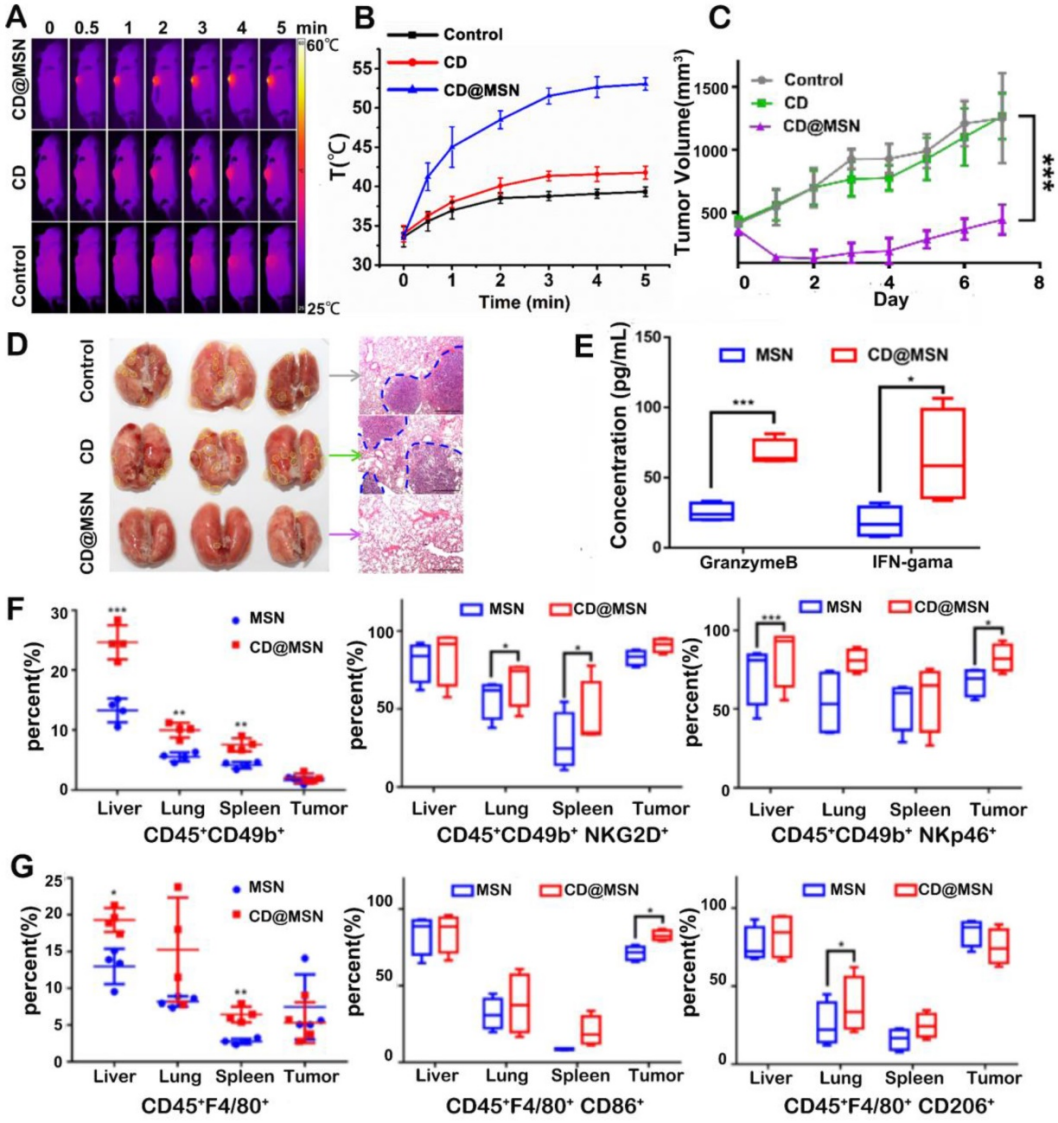
(A) Photothermal images and (B) tumor temperature curves of tumor-bearing mice after intravenous injection of CD or CD@MSN followed by 808 nm laser irradiation (0.75 W cm^-2^). (C) Changes of tumor volume over time after different treatments. Data were represented as mean ± SD (n = 4). Statistical significance was calculated by one-way ANOVA using the Tukey post-test (***p < 0.001). (D) Photos of the lungs harvested from different treatment groups 14 days after administration (the yellow dotted line circled the metastatic tumor focis) and corresponding H&E stained sections (the blue dotted line presented the border of the metastatic tumors), respectively. Bar = 100 μm. (E) Concentrations of granzyme B and IFN-γ in mice plasma from different groups. (F, G) Representative flow cytometric quantification of proliferation and differentiation of NK cells (F) and macrophages (G) gating on CD45^+^ cells harvested from different organs. Adapted with permission from 46, copyright 2019 Nano Letters.

Recent studies have shown that the hyperthermia generated by PTT can induce ICD, a favorable cell death phenotype that can attract immune cells and elicit robust immune responses 44. Cancer cell death triggered by hyperthermia can lead to the activation of danger signaling pathways and the release of DAMPs or TAAs, thereby markedly increasing the immunogenicity of those dying cells, and this kind of cell death pathway is termed as ICD. According to other reports, the induction of oxidative stress and endoplasmic reticulum (ER) stress play an essential role in this process 45. DAMPs are critical markers of ICD, including the expression of calreticulin (CRT), heat shock proteins (HSP70, HSP90) and the release of high mobility group box 1 (HMGB1) and ATP. They serve as danger signals to recruit immune cells and are crucial for DC maturation and antigen presentation. TAAs are antigen molecules that are overexpressed when normal cells become cancerous and also play important roles in activating immune responses. In an example reported by Qian *et al.* 46, the CD-incorporated mesoporous silica nanoparticles (CD@MSN) were prepared for combined photothermal immunotherapy. Herein, CD was incorporated into MSN to avoid rapid excretion and to improve the photothermal performances *via* CD condensation. After *i.v.* injection, the hybrid particles could perform PTT (*ca.* 55 °C within 5 min) on tumor cells upon NIR light irradiation and the hybrid particles could degrade into nanodebris due to CD-induced swelling (Figure 1A-B). Then the generated debris were able to capture the TAAs (H60 and mrn41 proteins) released from the dying tumor cells after photo-ablation and stimulate immune responses after extravasation from the necrotic tumor tissues into the nearby immune organs. As a result, the primary tumor growth was greatly retarded and lung metastasis was significantly inhibited (Figure 1C-D). It was also revealed that CD@MSN mediated PTT promoted the proliferation and activation of NK cells (Figure 1F) and increased the ratio of M1 (CD45^+^F4/80^+^CD86^+^) to M2 (CD45^+^F4/80^+^CD206^+^) type tumor-associated macrophages (Figure 1G). And the secretion of granzyme B and IFN-γ in plasma was also elevated (Figure 1E). However, the tumor inhibition in this study was not persistent probably due to insufficient immune activation by PTT alone. Moreover, despite the gradual degradability *in vitro*, the effect of CD@MSN on the physiological function of major organs should also be investigated.

In another example by Sweeny *et al.*, Prussian blue nanoparticles (PBNPs), an organic photothermal agent were designed to achieve combined photothermal-immunotherapy against neuroblastoma 47. Unlike other inorganic photothermal agents, PBNP is able to be degraded under physiological conditions of pH 7.4, which avoids causing potential toxicity. Moreover, PBNPs can efficiently convert NIR light into heat and raise the temperature at tumor site 48. Results showed that after* i.t.* injection, PBNPs mediated PTT could effectively eradicate the primary tumor for the neuroblastoma-bearing mice. Additionally, PTT could trigger ICD marked by the secretion of ATP and HMGB1 as well as elevated expression of CRT. After per-vaccination with PTT treated Neuro2a cells, mice were challenged with tumor cells again on the 7^th^ day. It was found that tumor progression became much slower and the long-term survival rate was also elevated. These results demonstrated that PTT could not only kill the primary tumors *via* hyperthermia but also trigger host immune responses to prevent the occurrence of tumors.

TAAs or DAMPs induced by PTT are a major source of antigens. In this case, antigens were generated after PTT and the combination therapy mainly aimed to eradicate the existing tumors and trigger immune responses to prevent tumor relapse or metastasis. However, there were also studies integrated the exogenous antigens with photothermal agents into one nanoplatform during preparation with aim to prevent tumor occurrence prophylactically. Such exogenous neoantigens could be ovalbumin (OVA), tumor cell derived exosomes or debris of tumor cell membrane, which can activate antigen-specific immune responses and arouse a long-term immune memory 39, 49.

**Figure 2:**
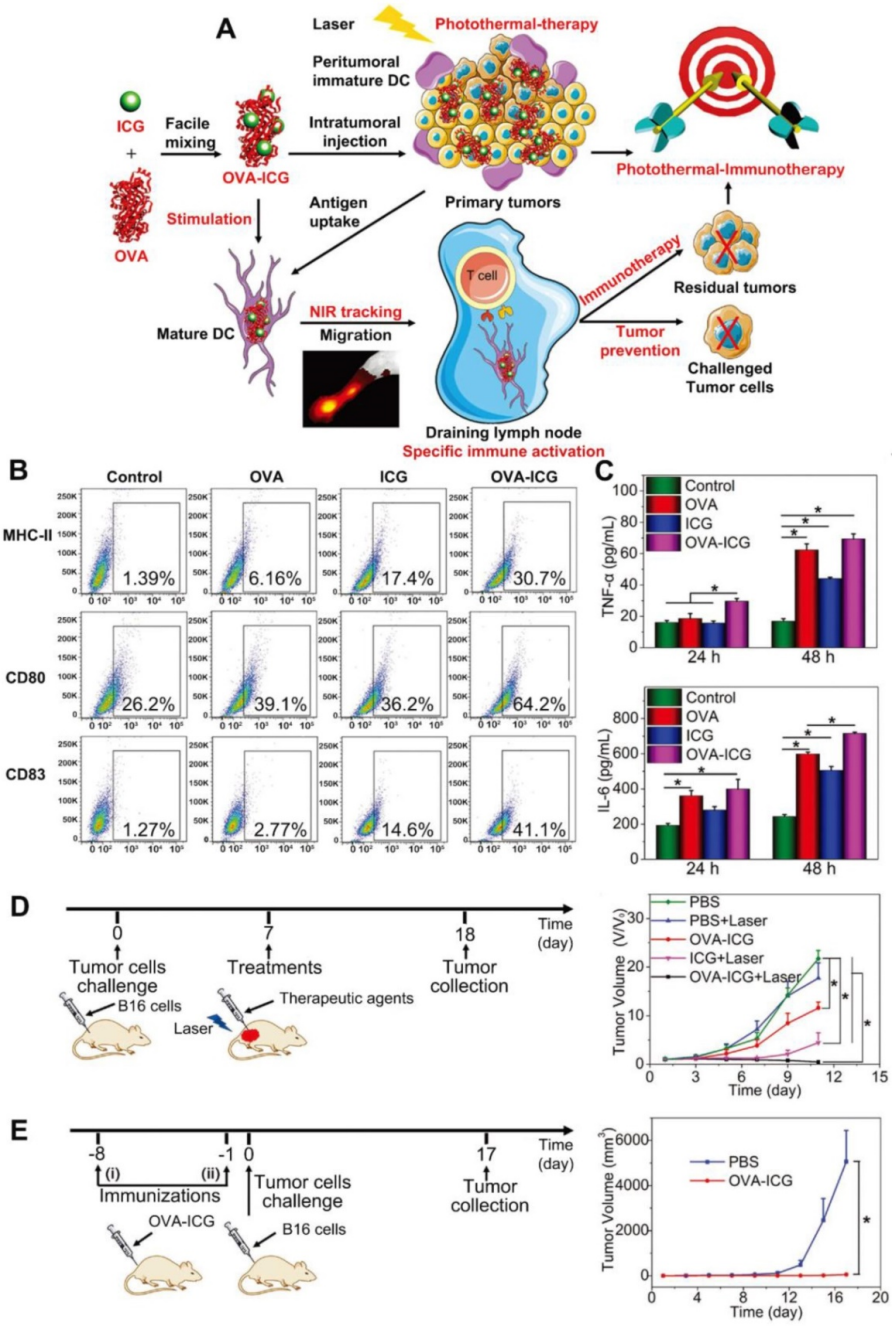
(A) Illustration for fabrication and mechanism of OVA-ICG nanovaccine for photothermal-immunotherapy against tumor, DC stimulation/tracking, and tumor prevention. (B) Expression levels of surface molecules (MHC-II, CD80, and CD83) on DC 2.4 cells after activation with pure OVA, pure ICG, and OVA-ICG nanovaccine. (C) TNF- α and IL-6 in the DC 2.4 cell culture supernatant after incubation with OVA, ICG, and OVA-ICG for 24 and 48 h. (D) Schematic illustration of protocol for therapeutic assay and tumor growth curves of the mice with different treatments. (E) Schematic illustration of protocol for tumor prevention assay and tumor growth curves of the mice treated with PBS or OVA-ICG. Adapted with permission from 50, copyright 2018 Advanced Materials.

For example, Pan *et al.* 50 utilized OVA both as a model antigen and a carrier to load ICG (Figure 2A) for combinational therapy. As one kind of organic small molecular dyes, ICG is a promising candidate for cancer theranostic application owing to its proved safety in clinic, ability for NIR fluorescence imaging and PTT. To improve its photo-stability and tumor accumulation, ICG was usually loaded in nanocarriers 51. This nanovaccine which had high antigen-loading efficiency (80.8%) could exhibit therapeutic and prophylactic dual functions. After treating immature DCs with ICG-OVA, the expression levels of MHC-II, CD80, and CD83 were significantly elevated. This indicates the activation and maturation of DCs (Figure 2B). Additionally, the levels of tumor necrosis factor-α (TNF-α, one marker in cellular immunity) and IL-6 (one marker in humoral immunity) in the cell culture medium were significantly increased (Figure 2C). After a single intratumoral injection of OVA-ICG followed by 808 nm laser irradiation, the tumor temperature increased to 60 °C, and the growth of B16 tumors was strongly suppressed. The anti-tumor efficacy of OVA-ICG plus NIR irradiation was much higher compared to that of single PTT mediated by ICG, which demonstrated the enhanced therapeutic efficacy of combined PTT and immunotherapy (Figure 2D). In addition, the population of CD8^+^ cytotoxic T cells in tumors and the anti-OVA immunoglobulin G (IgG) levels in the serum also increased. To test the prophylactical effect of this nanovaccine, mice were immunized with OVA-ICG twice before challenging with B16 cells. It was found that tumors barely grew, indicating its effectiveness in tumor prevention (Figure 2E). Also, exosomes which are a set of vesicles secreted by many cells play important biological roles in intercellular communication, body function maintenance and disease emergence. Since tumor cell derived exosomes, which carried tumor-specific antigens, could stimulate immune responses, they were explored to combine with PTT. In the study by Liu and co-workers 52, exosomes obtained from tumor-bearing mice after PTT (hEX) were used as tumor antigens and carriers for black phosphorus quantum dots (BPQDs). In this nanoplatform (hEX@BP), hEX could protect BPQDs, the photothermal agents from rapid degradation and clearance. What's more, hEX could target to tumors* via* their inherent homing ability. Lastly, hEX carrying tumor antigens was able to trigger immune responses. Mice were immunized with this vaccine every week for 3 times before *s.c.* inoculation of LLC (Lewis lung cancer) cells. From the results, hEX@BP immunization only inhibited tumor growth partially, indicating that immunotherapy was insufficient to completely prevent tumor occurrence. But once it was followed by BP mediated PTT after *i.t.* injection, tumor growth was significantly delayed, which again confirmed the improved therapeutic outcome of combinational therapy. However, exosome isolation which consumes lots of time and energy could be a challenge.

**Figure 3:**
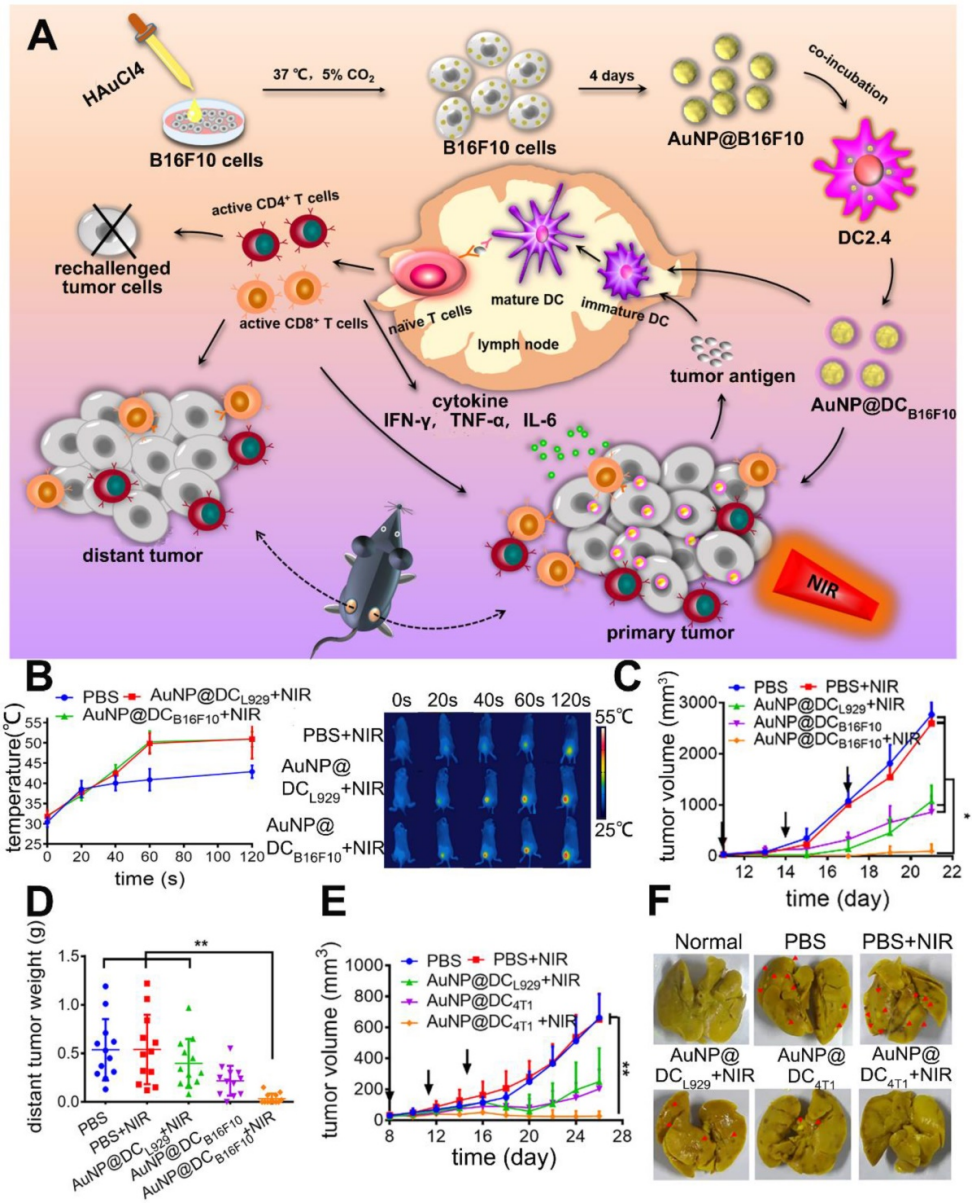
(A) Schematic preparation of AuNP@DC_B16F10_ and mechanism of AuNP@DC_B16F10_-mediated combinational treatment modality. (B) IR thermal images and corresponding temperature profiles of tumor-bearing mice injected with PBS, AuNP@DC_L929_, and AuNP@DC_B16F10_, respectively, with laser irradiation (n = 3). (C) Tumor growth curves after different treatments with or without laser irradiation of 2.0 W cm^-2^ (n = 7). The arrows represent the injection time. (D) Weight of the distant B16F10 tumor harvested at 19th day. (E) 4T1-tumor growth curve (n = 8). (F) Representative images of stained lungs of different groups. The arrows refer to the 4T1-tumor nodules on surface. Adapted with permission from 53, copyright 2019 Nano Letters.

In another article, Zhang's group prepared an immunological Au nanoparticle (Au NPs) *via* intracellular generation and exocytosis for combined PTT and immunotherapy 53. Briefly, Au NPs generated within B16F10 cells were trapped in vesicles and secreted into extracellular environment with tumor specific antigens (AuNP@B16F10). To further improve the immunological property and avoid potential metastasis caused by tumor derived vesicles, the AuNP@B16F10 was introduced to DCs and exocytosed as DCs derived vesicles (AuNP@DC_B16F10_, Figure 3A). After subcutaneous injection, this nanoparticle could gradually migrate to tumor and LNs over time. And the tumor temperature reached *ca.* 50 °C within 1 min under NIR light, which was high enough to induce tumor ablation (Figure 3B). Moreover, it was confirmed that AuNP@DC_B16F10_ carrying DAMPs and neoantigens from the B16F10 cells could be identified by the immune cells to trigger anti-tumor immunity. The anti-tumor effect was investigated on murine melanoma model and treatments were given for 3 times with 3 days interval. To elucidate the role of tumor antigen in inducing anti-tumor immunity, AuNP@DC_L929_ without transferred tumor antigen was prepared as control. In comparison to AuNP@DC_L929_ + NIR and AuNP@DC_B16F10_ which represented single PTT and single immunotherapy, AuNP@DC_B16F10_ + NIR achieved nearly complete tumor elimination with inhibition rate of 96.7% (Figure 3C). Moreover, AuNP@DC_B16F10_ + NIR could also significantly inhibit distant tumors (Figure 3D) and prevent tumor recurrence due to the effective activation of the CTLs, including the CD8^+^CD69^+^, CD8^+^CD107^+^ and CD8^+^ Granzyme B^+^ T cells in tumors and LNs. Of note, by replacing B16F10 cells with 4T1 breast cancer cells, the obtained AuNP@DC_4T1_ displayed similar anti-tumor effect on the 4T1 tumor models (Figure 3E-F). However, the clinical use of AuNP@DC_B16F10_ still faces challenges like the complicated procedure of preparation as well as difficulty in scale-up production.

## Nano-based PTT combined with immune adjuvant for tumor treatment

Although PTT alone can trigger host immunity, the efficacy is relatively weak. Therefore, immune adjuvants are often employed to combine with PTT for enhanced immune stimulation. PTT-induced DAMPs and TAAs in conjunction with immune adjuvants can form *in situ* vaccines. These are able to prime strong immune responses and induce long-term immune memory. Commonly used immune adjuvants are mainly Toll-like receptor (TLR) agonists, including cytosine-phosphate-guanine oligodeoxynucleotides (CpG ODNs, a TLR-9 agonist), resiquimod (R848, a TLR7/8 agonist), polyinosinic:polycytidylic acid (poly I:C, a TLR-3 agonist), and monophosphoryl lipid A (MPLA, a TLR-4 agonist); these are able to imitate the pathogen associated molecular patterns (PAMPs) to promote DC maturation and increase the excretion of immune-related cytokines 54.

**Figure 4:**
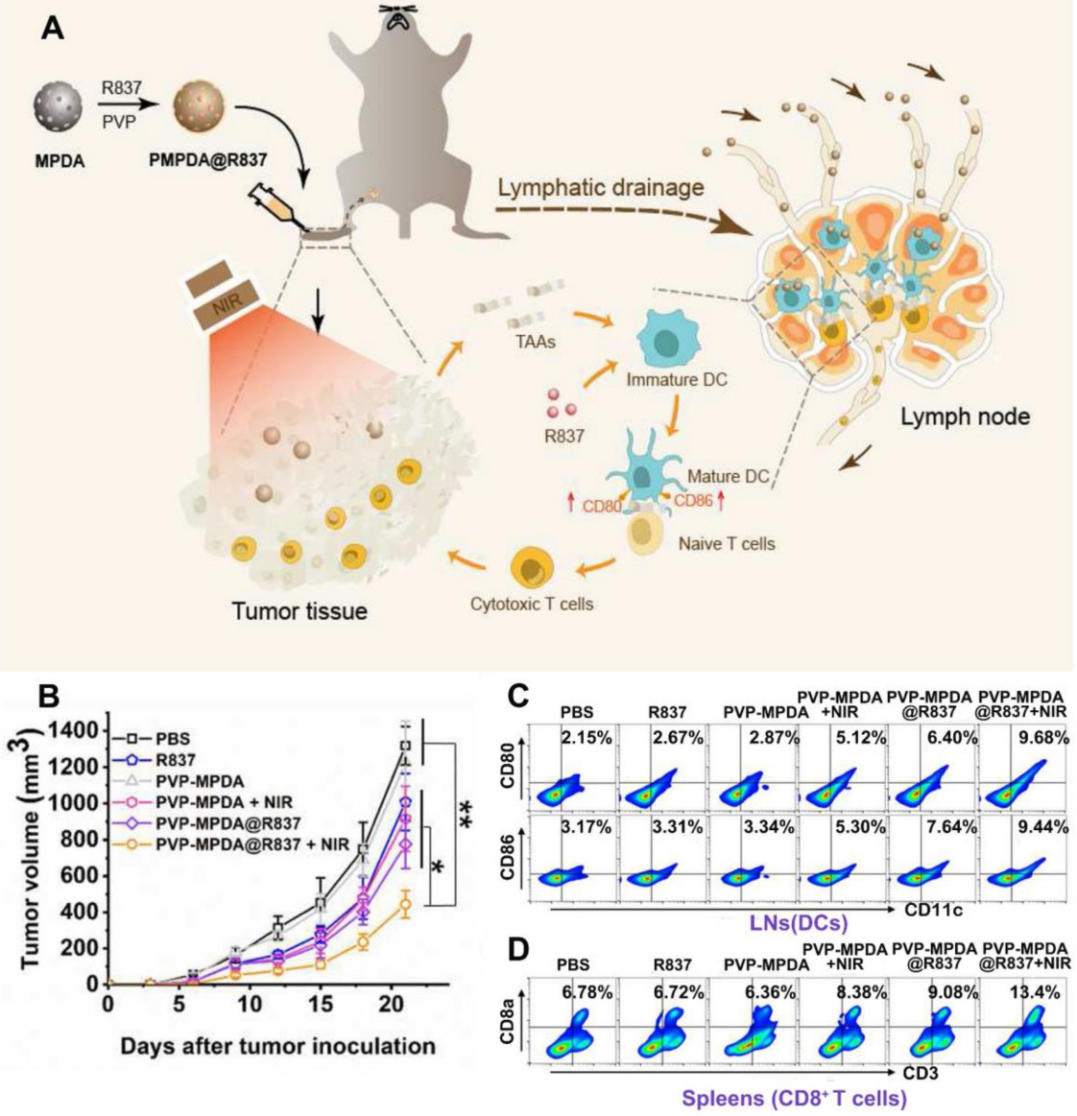
(A) Schematic depiction of PVP-MPDA@R837 nanoparticles and the mechanism of intrigued anti-tumor immune responses. (B) Tumor volumes of B16F10-bearing mice receiving different treatments. Footpad injections of various formulations were performed once every 3 d from 0 d for three times and tumor laser irradiation was applied at 24 h after injection. (C) Representative flow cytometry plots of CD80^+^ or CD86^+^ among CD11c^+^ DCs extracted from the popliteal LNs (tumor sentinel LNs). (D) Representative flow cytometry plots illustrated CD3^+^ CD8a^+^ T cells in splenocytes. Adapted with permission from 58, copyright 2020 Biomaterials.

TLR7/8 which is expressed on the intracellular compartments of many types of immune cells could recognize viral and bacterial RNA 55, 56. And TLR7/8 agonists such as R848 and R837 are synthetic imidazoquinoline-like molecules that function as immune modulators and possess anti-viral and anti-tumor activity 57. In the study by Wang and coworkers, R837 loaded mesoporous polydopamine nanoparticles (MPDA NPs) was prepared for combinational therapy (Figure 4A) 58. The synthesized NPs could encapsulate R837 with high efficiency owing to its porous structure. And after being coated with a PVP layer, these PVP-MPDA@R837 NPs could migrate to draining lymph nodes (DLNs) with long retention time. Consequently, drug exposure within the DLNs was greatly maximized and the off-target side effects of immunotherapy could also be alleviated. Herein, the MPDA acted as not only the carrier for the adjuvant R837 but also a photothermal agent with intrinsic biocompatibility and high photothermal conversion efficiency (up to 40%) 59. Results revealed that after peritumorally injection, the PVP-MPDA@R837 + NIR treated group exhibited the strongest inhibition effect on B16F10 tumor growth with an inhibition rate of *ca.* 66.3%, which was much higher compared to the PVP-MPDA + NIR (30.5%) and PVP-MPDA@R837 (41.2%) treated groups (Figure 4B). Moreover, the PVP-MPDA@R837 + NIR treatment also greatly elevated the maturation level of DCs in DLNs as well as the percentage of CD8^+^ T cells in the spleen of tumor bearing mice (Figure 4C-D). These findings confirmed the synergistic effect of PTT and immunotherapy. But whether the nanosystem could prevent tumor metastasis and recurrence was not investigated further.

R848 is also a commonly seen TLR7/8 agonist that outperforms R837 in promoting pro-inflammatory cytokine secretion and DC activation 60. In Chen's study 61, a conjugated polymer polyaniline-glycol-chitosan (PANI-GCS) was synthesized and loaded with R848 (denoted as R848@NPs) to induce mild hyperthermia and generate anti-tumor immunity. Herein, PANI in the polymer structure was used as the photothermal agent. PANI has been regarded as a favorable choice owing to its attractive properties including chemical stability, simple synthesis, as well as good optical property among the organic photothermal agents. It could induce a stable and strong hyperthermia effect due to the extended π-electrons 62, 63. However, the potential cytotoxicity of intermediates during the polymerization reaction of PANI could not be neglected, as reported by several studies 64, 65. In this study, hyperthermia was deliberately maintained at a relatively low temperature (*ca.* 44 °C) to spare the immune cells from heat induced elimination. It was found that the mild hyperthermia caused by R848@NPs upon laser irradiation significantly up-regulated the expression of HSP70, a DAMP molecule on CT26 cells. R848@NPs could be easily uptake by DCs and induce their maturation. The *in vivo* anti-tumor efficacy of different formulations was tested on subcutaneous CT26 tumor models *via i.t.* injection every 7 days, with a total of 3 injections. Results showed that hyperthermia induced by empty NPs at high temperatures (approximately 55 °C) caused severe burns at the tumor site. However, tumor inhibition was only transient, indicating the limited efficacy of conventional PTT. Moreover, the mild hyperthermia (*ca.* 44 °C) or single R848 treated groups only delayed tumor growth, while the low-temperature hyperthermia plus adjuvant (R848@NPs/+NIR) treated group exhibited the strongest tumor recession and the highest survival rate within 30 days of observation. The most encouraging finding was that after re-challenging the surviving mice with CT26 cells on the contralateral side, the second tumor barely grew, suggesting that the R848@NPs/+NIR could effectively prevent tumor recurrence and help to establish a durable anti-tumor immune memory. The underlying immunological mechanism revealed that the infiltration of CTLs within tumor tissue and the secretion level of proinflammatory cytokine IL-6 significantly increased. Also, the population of MDSCs and the level of immunosuppressive IL-10 in the TME decreased markedly after the combinational therapy.

**Figure 5:**
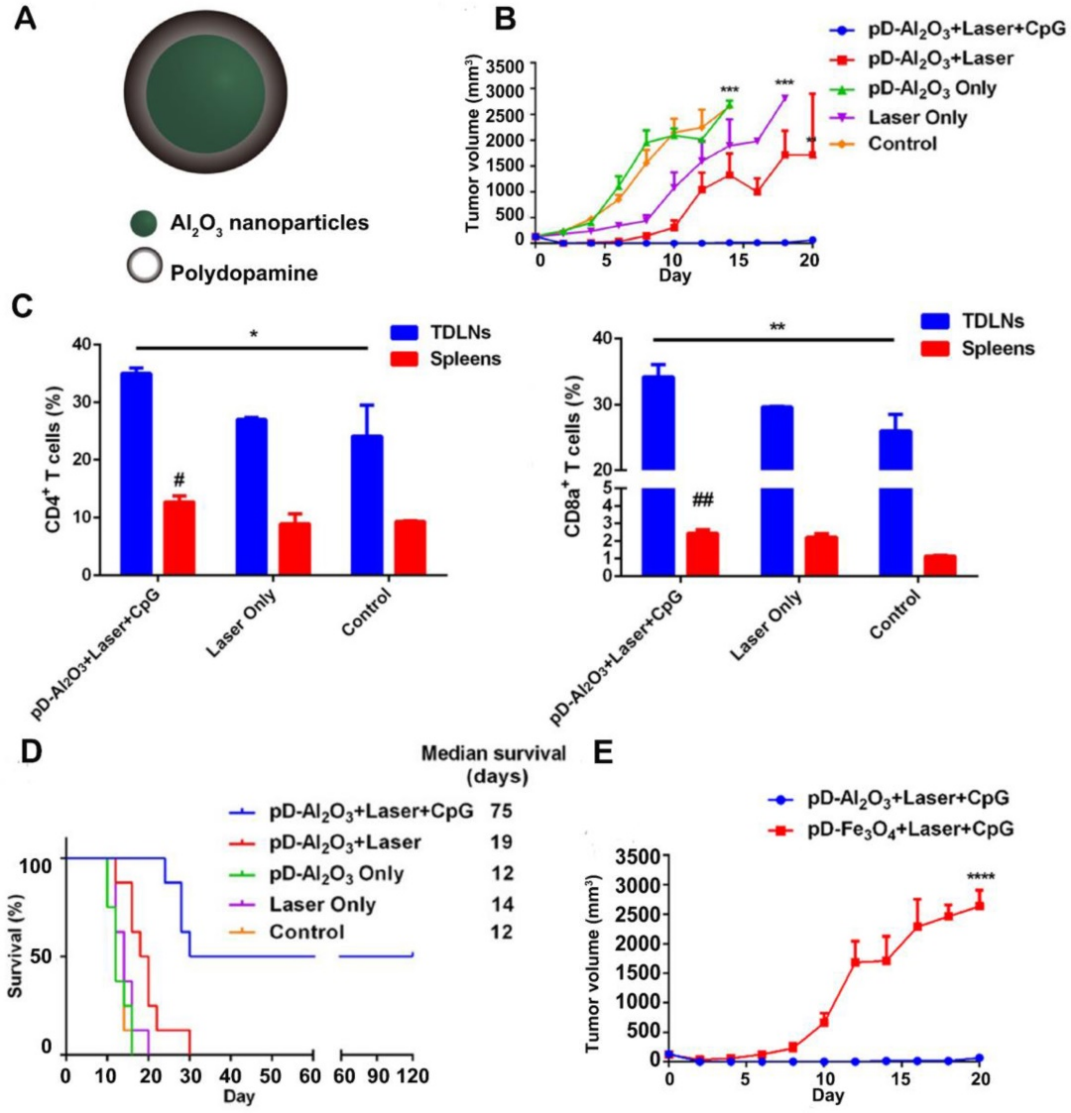
(A) Chemical structure of pD-Al_2_O_3_ nanoparticles. (B) Tumor growth curve after the indicated treatments (8 mice per group). (C) Proportions of tumor-infiltrating CD8^+^ T cells and CD4^+^ T cells in TDLNs and spleens of mice after the indicated treatments. Proportions were determined using flow cytometry. Pound signs in panel C indicate P values for comparisons between pD-Al_2_O_3_ + Laser + CpG and Control using the Student's 2-tailed *t* test. * P < 0.05; **P < 0.01. (D) Survival of mice after the indicated treatments (8 mice per group). (E) Tumor growth curve (8 mice per group). Adapted with permission from 67, copyright 2018 Theranostics.

Apart from small molecular adjuvants, CpG ODNs were also used as immune adjuvants in combination with PTT. Unmethylated CpG ODNs that bind to TLR9 can be efficiently internalized by APCs and stimulate the release of various cytokines, thereby initiating a series of innate and adaptive immune responses 66. For example, Chen and coworkers prepared pD-coated Al_2_O_3_ nanoparticles (pD-Al_2_O_3_ NPs) in combination with CpG for synergistic photothermal-immunotherapy. Herein, the pD functioned as a photothermal agent, and the inner Al_2_O_3_ core together with CpG served as an immune adjuvant 67 (Figure 5A). The results revealed that pD-Al_2_O_3_ NPs + CpG could induce the maturation of DCs both *in vitro* and* in vivo*. After intratumoral injection of pD-Al_2_O_3_ NPs, the temperature of the B16F10 tumor reached 55 °C within 5 min under NIR light. However, PTT alone failed to restrain tumor growth completely. When PTT was followed by *s.c.* injection of CpG for 3 times, tumors gradually shrank and became undetectable by day 18, demonstrating the importance of CpG in triggering strong anti-tumor responses (Figure 5B). Additionally, the combination of pD-Al_2_O_3_ NPs and CpG led to higher levels of CTLs (CD8^+^/CD3^+^) and helper T cells (CD4^+^/CD3^+^) in the tumor draining lymph nodes (TDLNs) and spleen (Figure 5C). The mice received combinational treatment survived for more than 75 days, revealing effective inhibition on tumor recurrence and metastasis (Figure 5D). Interestingly, the researchers also tried replacing the Al_2_O_3_ core with Fe_3_O_4_ but found tumor inhibition became less effective (Figure 5E). This indicated that the Al_2_O_3_ core and CpG dual played adjuvant functions in this system. There were also other studies using the IR820-conjugated hydrogels loaded with CpG self-crosslinked NPs 68 or the IR7 encapsulated liposomes coated with HA-CpG 69 for combined PTT and immunotherapy.

**Figure 6:**
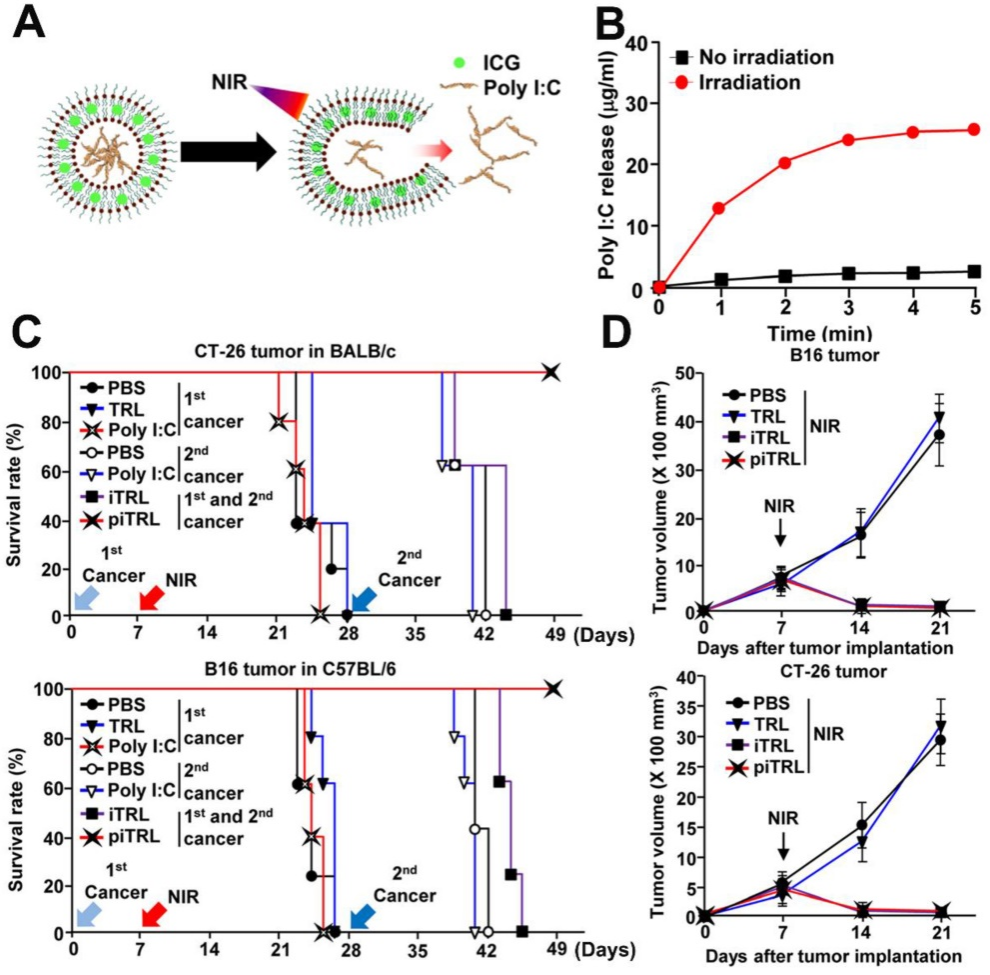
(A) Schematic diagram of poly I:C release from piTRL under NIR-laser irradiation. (B) The concentration of released poly I:C from piTRL under NIR-laser irradiation at a power intensity of 1W cm^-2^. (C) The survival rate of CT-26 challenged BALB/c mice and B16 challenged C57BL/6 mice were monitored, n = 5 for each group. (D) Tumor growth curves for CT-26 and B16 carcinoma with or without laser irradiation. Data are from the analyses of six individual mice. Adapted with permission from 72, copyright 2019 Journal for Immunotherapy of Cancer.

Additionally, Poly I:C is a double stranded RNA which binds to the TLR-3. It favors the cross-presentation of exogenous antigens by APCs 70 and is able to trigger both cellular and humoral immunity 71. Documented by Xu *et al.* 72, ICG and poly I:C co-loaded thermoresponsive liposomes (piTRLs) were prepared (Figure 6A) for combinational therapy. In this research, ICG based PTT could not only ablate cancer cells but also trigger the release of poly I:C from piTRLs to activate DCs in the TDLNs (Figure 6B). After intratumoral injection of piTRL into the CT26 or B16F10 tumor-bearing mice followed then by laser irradiation, the tumor temperature rose to *ca.* 52 °C, and tumor growth was almost completely inhibited. Then, the cured mice were re-challenged with tumor cells through *i.v.* injection. Results showed that single PTT treated mice all died after the second round of inoculation, probably due to the insufficient activation of immune responses by PTT. On the contrary, the piTRL + laser treated mice all survived, suggesting a key role poly I:C played in protection against the disseminated metastatic tumors (Figure 6C-D). Further experiments proved that such protection originated from the tumor antigen specific immune responses.

**Figure 7:**
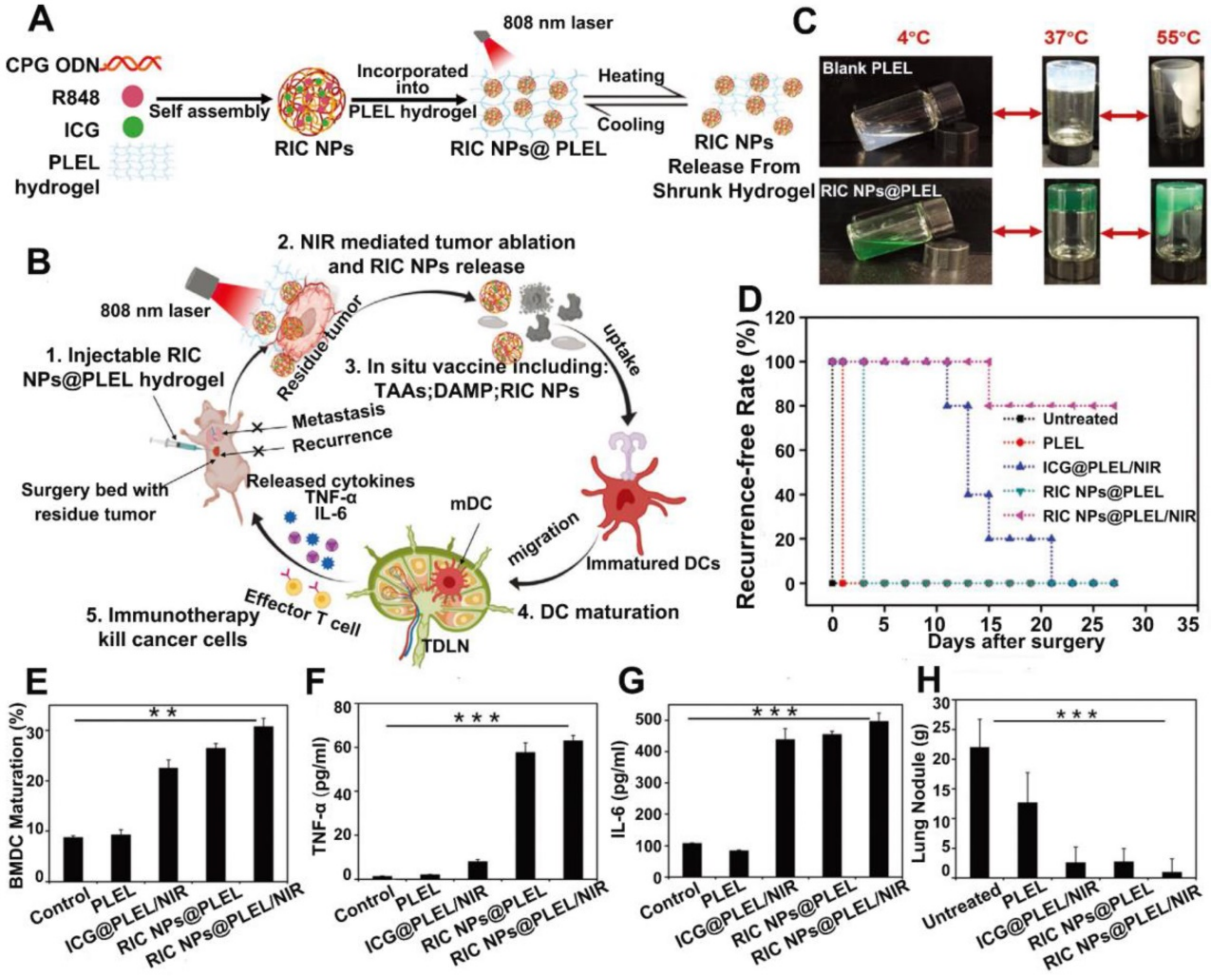
Schematic illustration of A) the preparation process of RIC NPs@PLEL hydrogels and B) photothermal-immunotherapy to prevent post-surgery tumor recurrence. (C) Reversible sol-gel transformation photos of PLEL hydrogels and RIC NPs@PLEL hydrogels. (D) Recurrence-free rate of mice after various treatments. (E) Maturation of BMDCs. (F, G) TNF-α and IL-6 secreted in the BMDC cell culture supernatant after different treatments. **p < 0.01 and ***p < 0.001. (H) Lung metastatic nodules at the end of different treatments. Adapted with permission from 76, copyright 2020 Advanced Functional Materials.

Recent studies also revealed that certain combinations of TLR agonists could strengthen the immune activation. Since different TLR agonists act on different PRRs, combination of them might mimic a natural infection and improve the antigen-specific immune responses 73, 74. For example, the combination of aluminum salt with a TLR-4 agonist, MPLA, was approved by FDA for usage in the human papillomavirus (HPV) vaccines 75. Therefore, dual immune adjuvants in combinatioan with PTT was also explored to further strengthen the anti-tumor immunity. Jia *et al.* 76 prepared a thermosentive PELE hydrogel loaded with RIC NPs which was self-assembled by ICG, R848 and CpG (RIC NPs@PLEL). This designed platform could act as an *in situ* vaccine to prevent postsurgery recurrence and metastasis (Figure 7A-B). The PLEL hydrogel was in liquid form at temperatures below 4 °C but quickly transformed into gels at body temperature, which greatly prolonged the retention of loading cargos at the injection site (Figure 7C). After incubation with 4T1 cells pretreated with RIC NPs@PLEL/NIR, DCs were successfully activated marked by an increased population of mature DCs (CD11c^+^CD80^+^CD86^+^) and promoted secretion of TNF-α and IL-6 (Figure 7E-G). After tumor resection, RIC NPs@PLEL was injected at the surgical bed followed by three cycles of laser irradiation (808 nm, 5 min), and 80% of the mice were completely cured without recurrence and no metastatic nodules were found in the lungs. In contrast, tumors all relapsed for other treatment groups, indicating the effectiveness of combinational therapy mediated by RIC NPs@PLEL (Figure 7D and 7H). In another similar study, surgically removed tumor cell membrane coated BPQDs (BPQDs-CCNVs) were loaded into a thermalsensitive hydrogel containing dual adjuvants for combined PTT and immunotherapy. BPQDs are biodegradable photothermal agents with high photothermal conversion efficiency and minimal cytotoxicity 28. Lipopolysaccharide (LPS) together with granulocyte-macrophage colony stimulating factor (GM-CSF) served as adjuvants 23. After firstly removing tumors by surgery, the Gel-BPQD-CCNVs were subcutaneously injected on the contralateral side and irradiated with NIR light. Tumor relapse and metastasis could be greatly inhibited. Results of flow cytometry revealed that the percentage of mature DCs in DLNs and the population of T cells in relapsed tumors were all markedly elevated. However, there was still a lack of explanation and discussion on the pairing of adjuvants and comparison between single adjuvant *vs.* dual adjuvants on the anti-tumor effect in these studies. Although these TLR agonists have demonstrated promising prospects and some of them have been evaluated in preclinical or clinical trials, there are still several challenges need to be addressed, including instability and off-target related side effects. For example, poly I:C could be degraded by serum nucleases, which hampers its capability in inducing IFNs or mediating anti-tumor immunity in primates. And there were reports about the severe side effects caused by R837, after oral or systemic administration 77. What's more, the adjuvanticity of different TLR agonists have not been compared. Further investigation is still needed to evaluate the efficacy and safety of these adjuvants.

**Figure 8:**
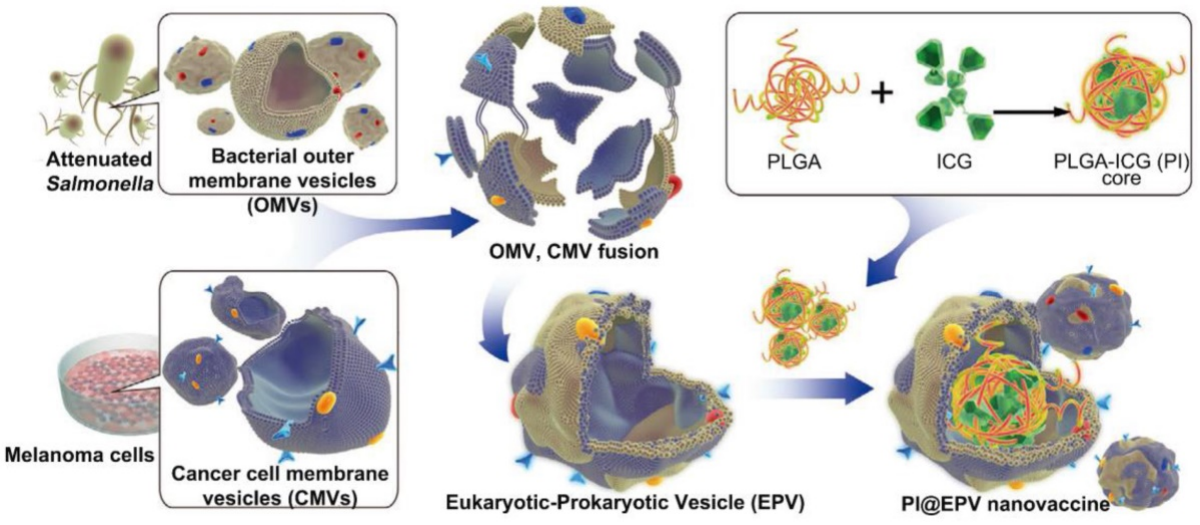
Schematic illustration of fabrication of eukaryotic-prokaryotic vesicles coated PI@EPV nanovaccine. Adapted with permission from 78, copyright 2020 Advanced Materials.

The integration of PTT with adjuvants could also be achieved using a biomimetic approach. For example, Chen *et al.* have prepared a eukaryotic-prokaryotic vesicle (EPV) nanoplatform by fusing melanoma cytomembrane vesicles (CMVs) with attenuated Salmonella outer membrane vesicles (OMVs) 78 (Figure 8). Herein, CMVs provided melanoma antigens and OMVs played the role of natural adjuvants. To further potentiate the tumor elimination capability, PLGA loaded with ICG(PI) was encapsulated into EPV (PI@EPV) to synergize immunotherapy with PTT. *In vitro* experiments found that PI@EPV was capable of stimulating DC maturation and T cell proliferation. And such immunoactivation was further boosted upon NIR laser irradiation due to the ICD effect caused by PTT. To test the ability of PI@EPV to prevent tumorgenesis, mice were intradermally immunized with nanovaccine for 3 times before the B16 or 4T1 tumor challenge. The PI@EPV treated group showed the highest tumor suppression rate (78.57%) as well as the longest tumor free time (19 days post-challenge) on the B16 tumor model. But it had no obvious suppression on the 4T1 tumor, revealing the specificity of anti-tumor immunity of melanoma cell vesicle-derived vaccine. Also, the PI@EPV vaccination greatly increased the tumor specific CTLs (CD3^+^CD8^+^CD107a) in spleens and enhanced the induction of effector memory T cells (CD3^+^CD8^+^CD44^+^CD62L^-^). Aside from the prophylactic effect, PI@EPV plus laser irradiation could also effectively eradicate the existing tumor with 80% of the treated mice became tumor free. Taken together, these results once again confirmed the enhanced anti-tumor efficacy after combining immunotherapy with PTT.

## Nano-based PTT combined with immune checkpoint inhibitors for tumor treatment

The recurrence and metastasis of tumors could not be well controlled by PTT alone. Although PTT assisted with immune adjuvants could increase tumor immunogenicity and boost immune activation, the ITM may still dampen the final therapeutic outcomes. The ITM is associated with the PD-1/PD-L1 interaction between T cells and tumor cells as well as the presence of some negatively regulating immune cells, including Tregs, MSDCs or M2 phenotype macrophages. Therefore, it is sometimes necessary to utilize immune checkpoint inhibitors or regulators to reverse the ITM in order to improve the therapeutic efficacy of PTT. At present, the PD-1 or PD-L1 antibodies (*e.g.,* pembrolizumab) 79 and the CTLA-4 antibodies (*e.g.,* ipilimumab) 80 are most widely used to reinvigorate the CTLs.

PD-1 is a type of immunosuppressive molecule expressed on the surface of many types of cells, including T cells. And it can specifically interact with a transmembrane protein, PD-L1 which is overexpressed on many malignant tumors 81. The binding of PD-L1 to PD-1 transmits inhibitory signals which lead to restrained cytokine excretion as well as apoptosis of T cells 82. Therefore, ICB treatment was proposed by using PD-1 or PD-L1 antibodies to recover the function of T cells by blocking the PD-1/PD-L1 pathway.

In the study by Liang *et al.*, the PD-1 antibody (aPD-1) was chosen to combine with PTT to treat basal like breast cancer 83. Since this type of cancer has high mutation load and extensive lymphocyte infiltration, it is more likely to respond to ICB. Herein, BPQDs were photothermal agents and the erythrocyte membrane (RM) was coated on BPQDs (BPQD-RMNV) to achieve long blood circulation and high tumor accumulation. In the animal experiments, Balb/c mice were inoculated with 4T1-luc cancer cells on both flanks with an interval of 7 days. The first one represented the primary tumor and the second one represented the metastatic tumor. On the 8^th^ day post-inoculation, mice were given different treatments through *i.v.* injection and subjected to NIR light irradiation. Subsequently, aPD-1 were *i.v.* injected every 3 days for 4 times. From the tumor size and bioluminescence signals, the combinational therapy (BPQD-RMNVs + laser + aPD-1) completely restricted the tumor growth on both sides and achieved the highest survival rate which significantly outperformed the aPD-1 or PTT treatment alone. The above results manifested the potential of combined PTT and immunotherapy in treating original and metastatic tumors.

Apart from combining ICB with PTT in the dose regimen, immune checkpoint inhibitors and photothermal agents could also be incorporated into one nanosystem for combinational therapy. Luo *et al.* encapsulated the anti-PD-1 peptide (APP) together with hollow gold nanoshell (HAuNS) into PLGA nanoparticles (denoted as AA@PN). It was found that a slow and continuous release of APP from nanoparticles could be obtained within 40 days 84. After single *i.t.* injection followed by multiple times of laser irradiation, AA@PN + laser significantly inhibited the growth of both primary and untreated distant tumors on 4T1 or CT26 tumor models. Moreover, the survival rate maintained above 80% which was much higher compared to immunotherapy or PTT monotherapy. Notably, they also discovered that multi-dose APP treatment (0.2 mg per injection for 10 times) was much more effective in tumor inhibition compared to single APP treatment (1 time injection of 2 mg). This finding revealed that perdurable PD-1 blocking was very important to the efficacy of immunotherapy. Since AA@PN could release APP in a sustained manner, a better therapeutic effect was therefore obtained as a result of long-term PD-1 blocking. Also, the number of CD8^+^ CTLs and the production of IFN-γ in distant tumors were markedly increased after AA@PN + laser treatment. To further restrict the action site of ICB, there was another study used a LM^D^P peptide-conjugated Au@Pt NPs (Au@Pt-LM^D^P) for combinational therapy. The LM^D^P peptide was synthesized by connecting a hydrolysis-resistant D-peptide (^D^PPA-1, a PD-L1 antagonist) with a tumor-targeting peptide LyP-1 (CGNKRTRGC) *via* an MMP2-responsive linker (PLGVRG) 53. After *i.v.* injection, this nanoplatform could accumulate at the tumor site and achieve on demand release of ^D^PPA-1 induced by the abundant MMP2 in the TME. Such site specific delivery of immune checkpoint inhibitors could greatly minimize the unwanted side effects. Results showed that the inhibition rates for the primary and distal tumors after Au@Pt-LM^D^P plus laser treatment reached 100% and 88.52%, respectively. This demonstrated that the combination of PTT with PD1/PD-L1 blockage could effectively eliminate primary tumor and prevent tumor metastasis. Similarly, the enhanced therapeutic outcome was due to higher population of CD8^+^ CTLs in tumor and spleen as well as more secretion of pro-inflammatory cytokines, including IL-6, TNF-α, IFN-γ and IL-12p70.

Aside from the PD-1/PD-L1 pathway, CTLA-4, a receptor mainly expressed on Tregs, is another critical negative regulator of immune responses. It opposes the actions of CD28 mediated co-stimulation by competing for CD80 and CD86 85. Inhibitors that block the CTLA-4 signaling pathway can destroy the interaction between CTLA-4 and CD80/CD86, thereby facilitating the binding of CD28 with CD80/CD86 to strengthen the T-cell responses and induce T cell mediated tumor rejection 86. In a study conducted by Juliana *et al.*, PBNP mediated PTT was combined with anti-CTLA-4 checkpoint inhibition for the treatment of neuroblastoma 87. The PBNP was *i.t.* injected into neuro2a tumor-bearing mice and irradiated with NIR light. The PTT treated tumors rapidly shrank at the beginning but soon recurred. While the PBNP based PTT + anti-CTLA-4 treated group exhibited complete tumor regression, with a survival rate of 55.5% over 100 days post treatment, which was significantly higher than that of the groups receiving anti-CTLA-4 alone (12.5%) or single PBNP-based PTT (0%). Moreover, the PTT + anti-CTLA-4 treatment also provided effective protection against secondary tumor growth after re-challenging the surviving mice with Neuro2a cells.

**Figure 9:**
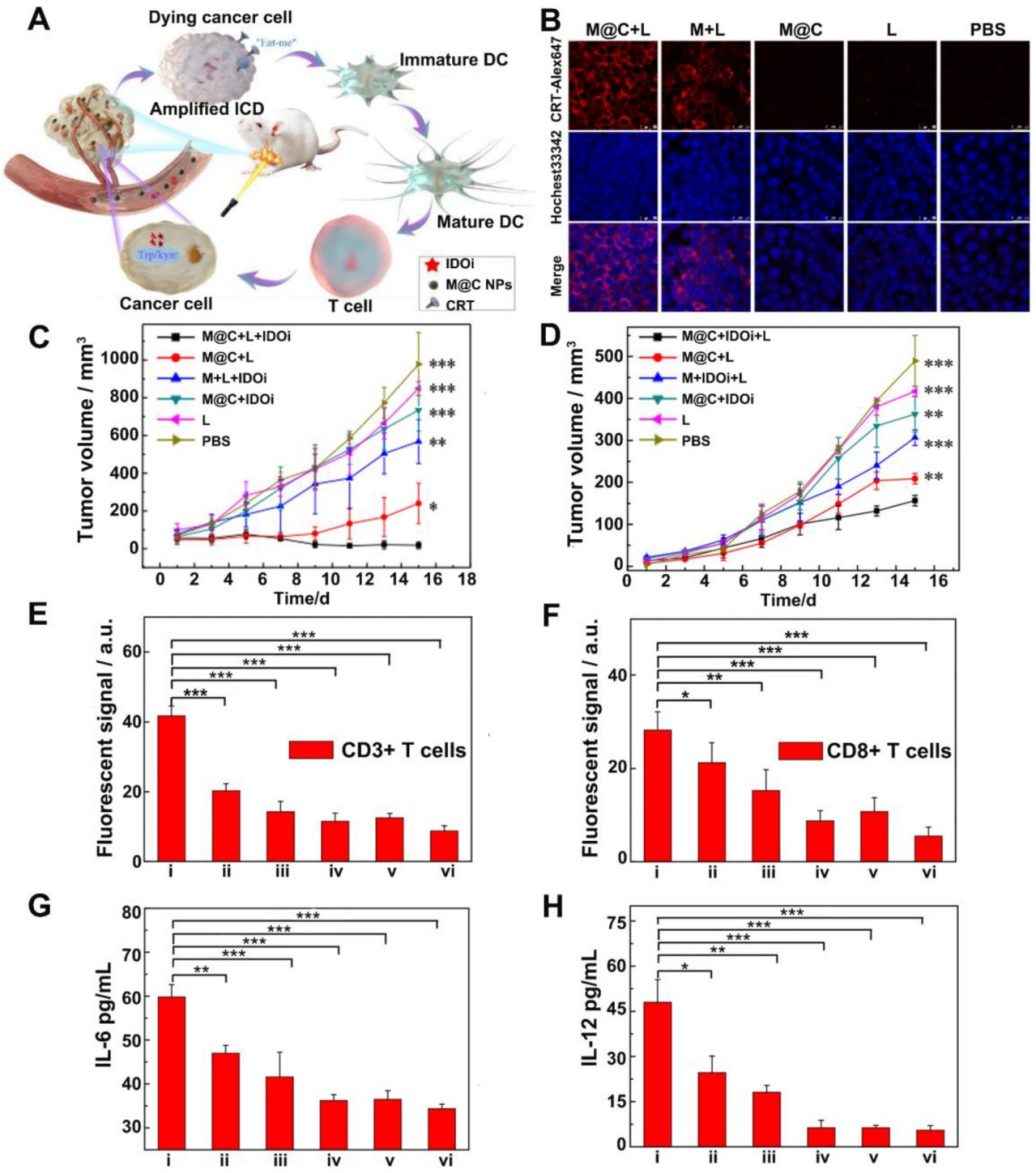
(A) Schematic illustration of M@C NPs for cancer immunotherapy by ICD induction and immune checkpoint blockade. (B) Expression of CRT proteins on the surface of 4T1 cells. (C) Changes of primary tumor growth. (D) The changes of distance tumors growth. (E) Quantification of fluorescent signals from immunofluorescent staining of CD3^+^ T cells and (F) CD8^+^ T cells in tumors. (G) ELISA analysis of IL-6 and (H) IL-12. i-vi represent mice treated with: (i)M@C + L + IDOi, (ii) M@C + L, (iii) M + L + IDOi, (iv) M@C + IDOi, (v) L, (vi) PBS. P values were calculated using the t-test (***P < 0.001, **P < 0.01, *P < 0.05) to compare other groups with group i. Adapted with permission from 90, copyright 2020 Chemical Communications.

Indoleamine 2, 3-dioxygenase (IDO) is overexpressed in the TME for many types of cancer. It has been found to be another potential immune checkpoint modulator that participates in the escape of cancer cells from immune surveillance during tumor progression 5. IDO is able to achieve enzymatic conversion of tryptophan to kynurenine, resulting in tryptophan deficiency, which disables the multiplication and functions of T lymphocytes and produces immune tolerance to tumor cells 88. Therefore, IDO inhibitors have drawn wide attention as another alternative for ICB to relieve ITM 89. For example, Li and coworkers 90 prepared cancer cell membrane-coated melanin nanoparticles (M@C NPs), in which melanin NPs served as photothermal agents with high photothermal conversion efficiency and good biocompatibility while the 4T1 cancer cell membrane helped the NPs evade clearance from circulation and target to the tumor site. Herein, the IDO inhibitor INCB24360 was used in combination with M@C NPs based PTT to enhance immune responses (Figure 9A). The results showed that PTT successfully induced ICD, as confirmed by the expression of CRT on the 4T1 cell membrane (Figure 9B). *In vivo* experiments revealed that the treatment with M@C NPs (*i.v.*) and IDO inhibitor (*i.p.*) plus laser irradiation strongly inhibited the growth of both primary and abscopal 4T1 tumors (Figure 9C-D). And the secretion levels of IL-12 and IL-6 as well as the percentage of CD3^+^CD8^+^ T cells within tumors were also the highest among all the treatment groups (Figure 9E-H). This indicated the synergistic effect of PTT and immunotherapy in provoking anti-tumor immune responses.

**Figure 10:**
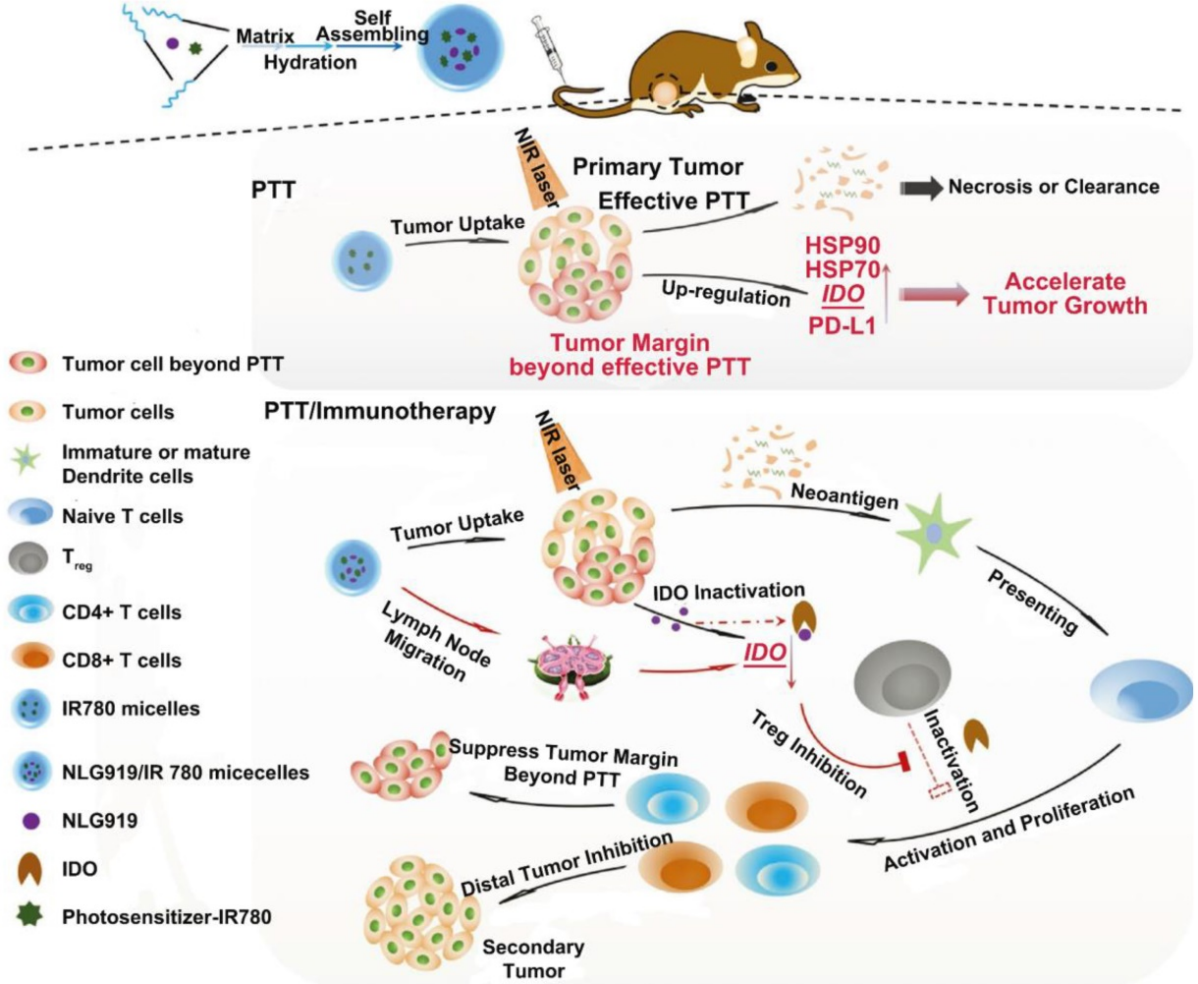
Scheme of the preparation pathway of NLG919(IODI)/IR780 co-loaded micelles and the mechanism by which the NLG919/IR780-micelle mediated PTT combined with immunotherapy suppressed the growth of the tumor margin beyond effective PTT and the distal (or secondary) tumor. Adapted with permission from 91, copyright 2018 Advanced Science.

In another study by Peng *et al.*, MPEG-PCL micelles co-loaded with an IDO inhibitor (NLG919) and a photosensitizer (IR780) were prepared for combinational therapy 91 (Figure 10). Researchers found that the penetration of PTT was rather limited. Around the heat generating source, the radius of tumor tissue with temperature above 43 °C was less than 6 mm. What's more, the tumor margin beyond effective PTT proliferated even faster due to the up-regulated HSP, PD-L1 and IDO which participate in the protection and evasion of cancer cells. Despite the enhanced tumor accumulation of micelles after *i.v.* injection, PTT mediated by IR780 micelles failed to restrain tumor growth compared to the control group; but once it combined with NLG919, significant tumor regression was observed. They also found that the combinational treatment could suppress the growth of secondary tumors as well as lung metastasis on 4T1 tumor models due to the inhibited IDO activity and elevated ratio of CD8^+^ T cells to Tregs.

**Figure 11:**
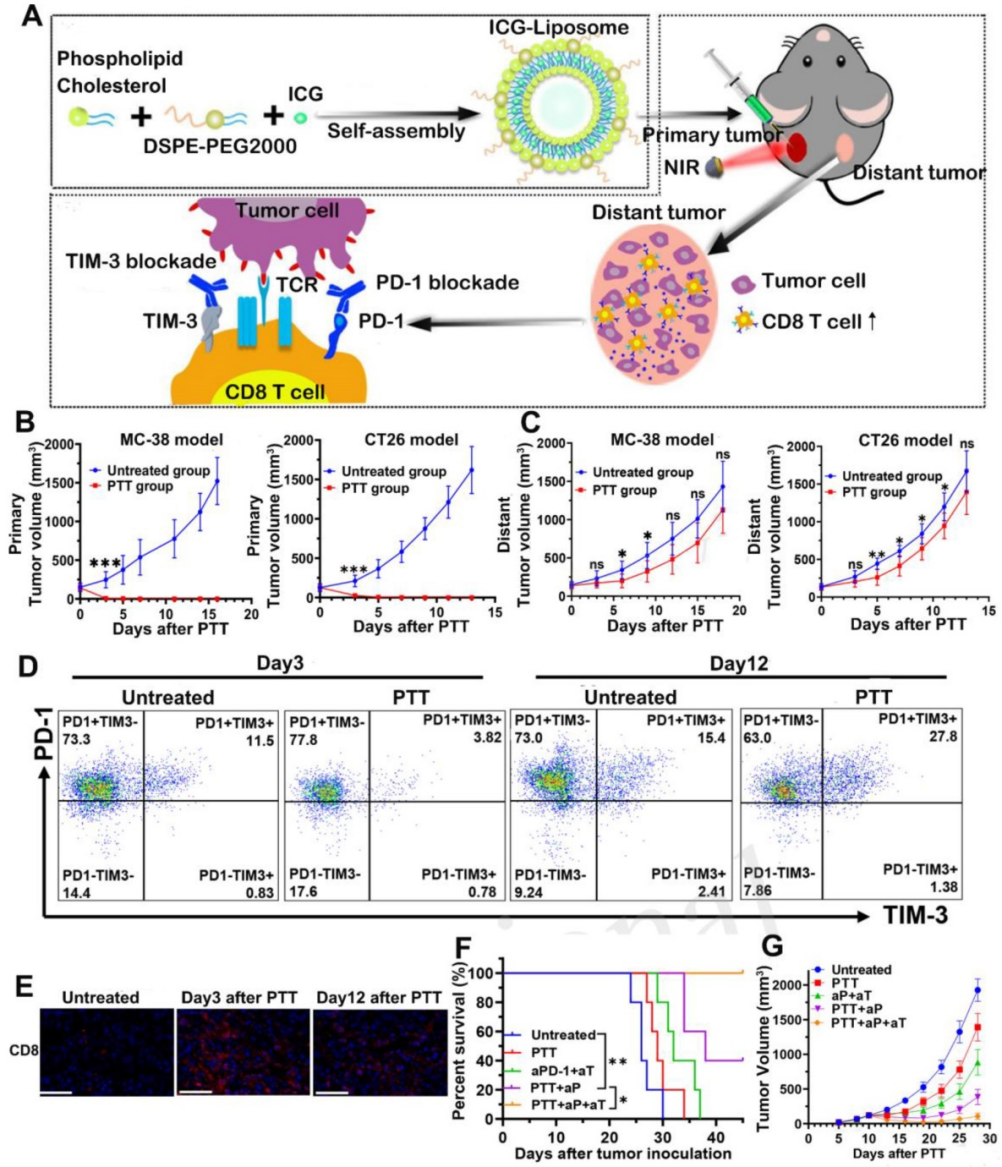
(A) The schematic illustration for ICG-loaded liposome as a theranostic nanoplatform and PTT in the primary tumor and immunological changes in the distant tumor after PTT with dual blockade of PD-1 and TIM-3. (B) Primary tumor growth profile of MC-38 and CT26 bearing mice from each group. (C) Distant tumor outgrowth curves of double MC-38 tumor-bearing mice and double CT-26 tumor-bearing mice. (D) Representative flow cytometric plots showing PD-1 and TIM-3 expression in tumor infiltrating CD8^+^ T cells on days 3 and 12 after PTT. (E) CD8^+^ T cells infiltration of each group in the MC-38 TME on day 3 and day 12 after treatment were examined by immunofluorescence. (F) Kaplan-Meier survival curves and (G) distant tumor growth of tumor-bearing mice. Adapted with permission from 93, copyright 2020 Frontiers in Chemistry.

Notably, there were also studies using two kinds of ICBs combined with PTT to enhance the therapeutic outcome. T cell immunoglobulin and mucin domain-containing protein 3 (TIM-3) is an indicator of antigen-specific T cell exhaustion and involves in the negative regulation of Th1 response. Therefore, anti-TIM-3 antibody has been used to relieve T cell anergy 92. For example, Huang *et al.* 93 prepared ICG encapsulated liposomes to perform PTT and combined it with dual PD-1/TIM-3 blockade for effective cancer treatment (Figure 11A). In this study, the anti-tumor efficacy was tested on the bilateral MC-38 and CT26 tumor models. From the results, local PTT raised the tumor temperature to near 60 °C and completely ablated the primary tumors (Figure 11B). PTT also exhibited modest inhibition on distant tumors at an early stage after treatment, which was due to the trafficking of activated CD8^+^ T cells from peripheral immune organs to tumor tissues (Figure 11C and 11E). However, PTT alone was insufficient to maintain long-term recession of distant tumors, since the CD8^+^ T cells gradually underwent functional exhaustion as a result of compensatory up-regulation of PD-1 and TIM-3 at a later stage after PTT (Figure 11D). After combining PTT with dual blockade of PD-1 and TIM-3 (PTT + aP + aT), the distant tumor growth was significantly delayed with a tumor-free survival of 40% (Figure 11F-G). Such superior anti-tumor efficacy proved the synergistic effect of PTT with immunotherapy.

Furthermore, the integration of ICBs, immune adjuvants and PTT was also explored to obtain a better therapeutic effect. For example, mPEG-PLGA polymer co-loaded with Fe_3_O_4_ superparticles and R837 (Fe_3_O_4_-R837 SPs) was prepared to combine with PD-L1 checkpoint inhibitor for the treatment of 4T1 murine breast cancer 94. Herein, the Fe_3_O_4_ SPs could not only perform PTT but also increase tumor accumulation *via* magnetic targeting. The *in vivo* results revealed that single PTT mediated by Fe_3_O_4_ SPs could directly eradicate the primary tumors but had little effect on the distant tumors. With the involvement of immune adjuvant R837, the inhibitory effect on distant tumors was modestly enhanced. When further combined with ICB, the Fe_3_O_4_-R837 SPs + anti-PD-L1 + laser showed significant inhibition on both the primary and distant tumors and effectively prevented lung/liver metastasis. And it is worth mentioning that ICB therapy alone could not well control the secondary tumor growth, which suggested that only when the tumors had turned from “cold” to “hot” after PTT and became immunogenic enough, the benefits of ICB therapy could be obtained. In another example, Song and coworkers have developed a PEGylated Bi_2_Se_3_ nanocage loaded with R848 (NC-PEG/R848) to combine with ICB 95. The Bi_2_Se_3_ NC possessed distinguished photothermal conversion ability, and the hollow structure favored R848 loading with high efficiency. And PEGylation facilitated the tumor accumulation of NC *via* EPR effect. Likewise, the combination therapy was the most effective in inhibiting the primary and distant tumors.

## Other strategies to synergize nano-based PTT with immunotherapy

**Figure 12:**
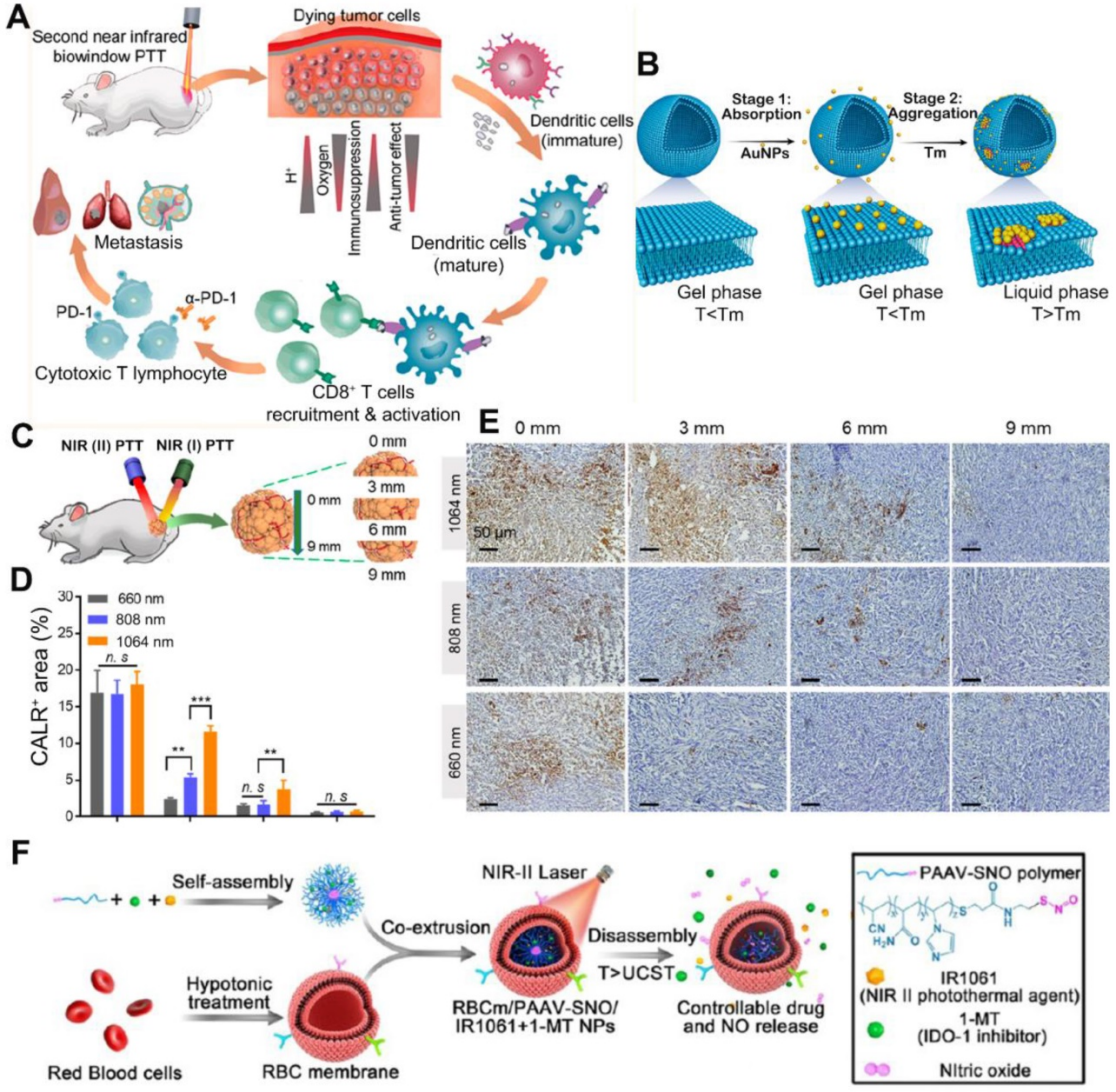
(A) Schematic illustration of NIR (II) PTT combined with immunotherapy for cancer treatment. (B) Schematic showing controllable aggregation of AuNPs on fluidic liposomes. Local DOPC clusterings were shown in red color. AuNPs were absorbed on gel-phased liposomes when the temperature was below melting temperature and aggregated when the temperature was above melting temperature. (C) Schematic diagram of the *in vivo* CALR exposure at different depths inside the tumor under the NIR(I) or NIR(II) laser irradiation. (D) Percentage of CALR positive areas of dissected tumor tissues at different depths (0, 3, 6 and 9 mm). Data are shown as mean ± SD (n = 3) **p < 0.005, ***p < 0.001, ****p < 0.0001. (E) Immunohistochemical staining of CALR exposure in 4T1 tumors received 660, 808, and 1064 nm PTT. (F) Schematic showing the structure and therapeutics releasing process of erythrocyte membrane-camouflaged nanobullets. Adapted with permission from 96, 97, copyright 2020 and 2019 ACS Nano.

Recently, the exploration of PTT in the second near infrared (NIR II, 950-1350 nm) window has drawn great attention. Compared to the first NIR light (NIR I 650-950 nm), the NIR II biowindow with minimal photo scattering, low tissue background, and deep tissue penetration depth to *ca.* 3-5 cm is more attractive for PTT 96. Photothermal agents which could respond to the NIR II light can induce more homogeneous and extensive ICD, and reverse the TEM from “cold” to “hot”. In the research by Ma *et al.,* citrate coated Au NPs with 40 nm were attached to the DOPC liposomes(Au_40C_-DOPC) to achieve the NIR II PTT, since the aggregation of Au NPs on the lipid membrane could lead to the redshift of LSPR as a result of interparticle plasmon coupling (Figure 12A-B). Compared to the light of 660 nm and 808 nm, the NIR II light of 1064 nm had deeper penetration depth within tumor tissues (Figure 12C-E). Upon irradiation by 1064 nm laser, Au_40C_-DOPC effectively induced ICD at the very inside of tumor and significantly elevated the infiltration of CD8^+^ T cells. To test the tumor prevention effect, mice were *s.c.* injected with 4T1 cells pre-treated by Au_40C_-DOPC mediated PTT twice with 1 week apart. After re-challenging the mice with live tumor cells at the 7^th^ day post vaccination, the tumor growth was significantly delayed with 5/8 of the mice remained tumor free. Researchers also tested the anti-tumor efficacy of NIR II PTT combined with immune adjuvant (R837 loaded in PEG-PLGA micelle) and immune checkpoint inhibitors (anti-PD-L1) on the bilateral tumor models. Results showed that both the primary tumor and distant tumor was suppressed significantly after the combinational therapy. Similarly, Yang and co-workers explored the anti-tumor effect of IR1061 mediated NIR II PTT combined with IDO inhibitor (1-MT) 97. IR1061 and 1-MT were co-encapsulated into nanoparticles self-assembled by a poly (acrylamide-co-acrylonitrile-co-vinylimidazole)-S-nitrosothiols copolymer, PAAV-SNO). Then the nanoparticles were camouflaged with an erythrocyte membrane for long circulation (Figure 12F). Herein, SNO is a thermal sensitive nitric oxide (NO) donor, which could release NO upon the breakage of S-NO bond by PTT 98. After further being camouflaged with an erythrocyte membrane, the final obtained nanoparticles were denoted as RBCm/PAAV-SNO NPs. From the bio-distribution study, the RBCm facilitated the tumor accumulation of nanoparticles due to its ability to avoid phagocytic clearance and extend blood circulation. The IR1061 mediated NIR II PTT could abate the tumor cells deeply and effectively induce ICD marked by increased CRT exposure and HMGB1 release. Upon 1064 nm laser irradiation, the release of NO and 1-MT within tumor tissue was triggered as a result of chemical bond cleavage and particle disassembly. Moreover, with the vessel normalization and hypoxia relief mediated by NO and the IDO inhibition by 1-MT, the ITM was efficiently reversed. To test the therapeutic effect, 4T1-tumor bearing mice were* i.v.* injected 3 times with different formulations and subjected to laser irradiation or not at 24 h post injection. Results revealed that single IDO inhibition only had limited anti-tumor efficacy, once combined with PTT and NO gas therapy, the tumor growth was significantly inhibited with 4/6 mice became tumor free. By analyzing the intratumoral cell population, an increase of tumor infiltration of CD8^+^ T cells as well as decreased number of Tregs and M2-like macrophages were found after the combinational treatment. Also, the level of TNF-α and IFN-γ was greatly augmented.

Other than immune adjuvants and ICB, CAR-T therapy is also an appealing option for combination with PTT. The principle of CAR-T therapy is that by modifying T cells with chimeric antigen receptors which specifically target to tumor, the tumor cells could be easily identified and eliminated by T cells without the involvement of major histocompatibility complex (MHC) 99. However, the desmoplastic structure of tumor and the presence of ITM usually attenuated the efficacy of CAR-T therapy. To overcome these challenges, Chen *et al.* combined the PLGA-ICG (PI) mediated PTT with CAR T therapy to obtain a better therapeutic outcome 100. In this study, CAR T cells which target to the chondroitin sulfate proteoglycan-4 (CSPG4) antigen overexpressed in melanoma (*e.g.,* WM115 cell line) were prepared. It was found that WM115 cells pre-treated by PTT could induce a vigorous proliferation of CSPG4.CAR T cells as well as an increased production of IL-2 and IFN-γ. Also, PTT was able to increase tumor perfusion, relieve the hypoxia and facilitate the accumulation of CAR CSPG4^+^ T in tumors on the WM115 xenograft model. The combinational anti-tumor activity was also verified. After *i.t.* injection of PI followed by laser irradiation (808 nm, 0.3W cm^-2^, 20 min), 1 × 10^7^ CAR.CSPG4^+^ T cells were *i.v.* injected. The combined treatment significantly inhibited tumor growth with 2 out of 6 mice being completely cured. This result confirmed the superior anti-tumor efficacy after combining PTT with CAR-T therapy.

Recently, the stimulator of interferon gene (STING) mediated immunotherapy has been focused on. STING is a kind of ER membrane signaling protein, which can sense cyclic dinucleotides (CDNs) and activate immune response as well 101. STING agonists have been exploited as new immune adjuvants. Several studies have shown that STING signaling is vital to anti-tumor immunity in triggering the production of type I IFN and activating both the innate and adaptive immunity 102. Zhao *et al.* have prepared a nano-complex composed of cyclic dimeric guanosine monophosphate-adenosine monophosphate (cGAMP, a STING agonist) and the polyethyleimine modified golden nanorod (GNR-PEI) for combined treatment 103. It was revealed that after co-incubation with the osteosarcoma K7 cells pre-treated by GNR-PEI/cGAMP with or without laser irradiation, the BMDCs was effectively activated *via* a STING dependent signaling pathway marked by the up-regulation of phosphorylated interferon regulatory factor 3 (IRF3), IFN-β and CXC chemokine ligand-10 (CXCL10). And the treatment of GNR-PEI/cGAMP + laser successfully elicited ICD evidenced by augmented CRT exposure, HMGB1 release, and ATP secretion. Even though GNR-PEI/cGAMP + laser could strongly inhibit the primary tumors on K7 xenograft model, it was insufficient in restraining the secondary tumor growth. After further complemented with anti-PD-1, the growth of distant tumors was significantly retarded. Also, an increased population of CD8^+^ T cells, M1-like macrophages and NK cells together with a decreased percentage of MDSC, Tregs and M2-like macrophages were observed within tumors.

Additionally, PTT combined with cytokine therapy for the oral treatment of tumors has been reported as well. Fan and co-workers 104 have creatively developed a thermally-sensitive programmable bacteria (TPB) based on the non-invasive bacterium, E. coli MG1655. The TPB was transformed with the plasmid pBV200 which contained a thermally-sensitive promoter TcI and the gene of a therapeutic protein, TNF-α. Then bio-mineralized Au NPs were decorated on TPB obtaining TPB@Au to achieve PTT assisted immunotherapy. Results showed that TPB could prevent the decomposition of loaded genes in stomach, enter systemic circulation from the gastrointestinal tract and target to tumor tissues due to their homing ability to TME. Upon laser irradiation, the heat generated by PTT relieved the TcI repression and initiated the expression of TNF-α to induce the apoptosis of tumor cells. Results revealed that the 4T1 tumors were significantly inhibited after the treatment by TPB@Au plus laser mainly as a result of intratumoral release of TNF-α. However, the anti-tumor effect of single PTT was not compared in parallel. Although it was shown that TPB@Au had negligible impact on intestinal microflora and homeostasis, the safety of this system still needs further confirmation. Similarly, in the study by Lin *et al.*, a MSN co-loading CuS within the pore and a plasmid encoding IL-12 gene on the outer surface (CSP@IL-12) was prepared for combinational treatment 105. IL-12 is a kind of pro-inflammatory cytokine mainly secreted by APCs and plays a vital role in the proliferation of CTLs and NK cells. It could also trigger the production of IFN-γ, TNF-α, IL-2, GM-CSF perforin and granzyme B to enhance the anti-tumor immune responses. From the results, the secretion of IL-12 by B16F10 cells could be detected after effective gene delivery by the CSP nanoparticles. Upon laser irradiation, the CSP@IL-12 significantly induced ICD on B16F10 cells and promoted the maturation of DCs. However, the influence of heat generation on the IL-12 gene expression was not investigated. Also, the CSP@IL-12 + laser showed the strongest inhibition on both the primary and distant tumors compared to single PTT or IL-12 cytokine treatment.

## Conclusions

Overall, nano-based PTT could effectively debulk primary tumors by thermal ablation and induce ICD to trigger host immune responses. However, increasing evidence suggests that PTT alone cannot control distant tumor growth and prevent metastasis. After combining PTT with immune adjuvants, the maturation of DCs was significantly promoted, as was the secretion of pro-inflammatory cytokines, which favors the activation and infiltration of CTLs to the tumor site. Also, the introduction of exogenous tumor antigens could further strengthen the immunogenicity of tumors. Based on the results of many research articles, immune adjuvant-assisted PTT showed stronger inhibition on both primary and distant tumors. A growing number of studies have begun to realize that the modulation of ITM is of equal importance compared to immune stimulation. PTT was alternatively combined with immune checkpoint inhibitors, such as anti-PD-1/PD-L1, anti-CTLA-4 or IDO inhibitors. Results showed that ITM was effectively relieved after the combined treatment, marked by an increased ratio of CTLs to immunosuppressive cells (Tregs, M2 phenotype macrophages or MDSCs) within tumor tissues. This combination was found to be effective in complete tumor elimination and metastasis prevention since those inhibitors could unleash the CTLs to kill tumor cells by shutting down the negative regulating pathways. Moreover, enhanced anti-tumor efficacy could also be obtained after combining PTT with CAR-T therapy or cytokine therapy. Since PTT was able to increase the tumor infiltration of immune cells and to control the release of immune activating cytokines. In view of limited penetration depth of NIR I PTT, photothermal materials which in the NIR II biowindow were also explored, and the combination of NIR II PTT with immunotherapy have achieved satisfied therapeutic effect on animals.

Until now, a variety of nanoplatforms have been developed. And they played important roles in the combination of PTT with immunotherapy, such as 1)serving as carriers to encapsulate photothermal agents and immune modulators, sometimes nano-sized photothermal agents could be carriers by themselves; 2)protecting the loaded cargo from rapid degradation or clearance; 3)enabling the therapeutic agents to accumulate within tumors or migrate to LNs to alleviate systemic side effects; 4)facilitating the cellular uptake of antigens and the controlled release of immune modulators. Apart from these capabilities, an ideal nanoplatform should also have good biodegradability and biocompatibility, which is the prerequisite for clinical use in the future.

In summary, the combination of nano-based PTT with immunotherapy has shown great potential and efficacy in fighting against cancer and more therapeutic strategies and nanomaterials are yet to be explored in order to obtain better therapeutic effect and benefit the patient clinically.

## Perspectives

Even though satisfactory therapeutic outcomes were obtained after combining PTT with immunotherapy in lots of studies, there are still several concerns that need to be addressed. Firstly, the optimal temperature for PTT remains to be confirmed. Conclusions from different articles were sometimes contradictory. In the example reported by Sweeney and coworkers 47, PBNP-mediated PTT within an optimal thermal window from 50 to 60 ℃ could trigger ICD more effectively and demonstrated a much higher survival rate on the neuroblastoma tumor model compared to that within the lower temperature range (<50 °C) or higher temperature range (>80 °C). And many studies introduced in this review achieved effective tumor ablation with PTT at temperatures above 50 °C. However, there were also studies emphasizing the importance of low-temperature hyperthermia. For example, many researchers 61, 106 argued that high temperature (>50 °C) would cause damage to normal issues, including immune cells, while mild hyperthermia (*ca.* 45 °C) was beneficial to induce DAMPs such as HSP70, which was necessary to trigger immune responses. Moreover, the slightly elevated temperature also facilitates the migration of various immune cells to the tumor site due to accelerated blood and lymph flow. Taken together, higher temperatures are favorable for tumor ablation but may adversely affect immune cells. While low temperature might be insufficient for tumor eradication, but it can modulate the local TME and better activate the immune system. Therefore, the optimal thermal dose remains to be explored in the future. Secondly, the administration form of the nanoplatforms also needs to be carefully considered. Hydrogels could be a good option for local drug delivery co-encapsulating photothermal agents and immune regulators in treatment of superficial tumors (*e.g.,* melanoma). And many studies applied the hydrogels on the surgical bed after tumor resection to eliminate the residual tumor cells 76, 107, 108. The biocompatible hydrogels with high loading efficiency can achieve controlled or sustained release of immune modulators. Moreover, hydrogels can prolong retention time of photothermal agents which can reduce the times of administration. For example, in Jia and coworkers' research 76, which was mentioned before, after one intratumoral injection of RIC NPs@PLEL on day 0, the tumor site could be repeatedly irradiated on days 1, 4 and 7, which was highly convenient. However, it is regretful that hydrogel so far was not suitable for systemic administration. Thirdly, the dose regimen or schedule varied greatly between studies, including the dosage of photothermal agents and immune modulators as well as the timing, frequency and route of administration. However, there were rare studies compared the effect of dose regimen on the anti-tumor efficacy of combinational treatment. And the optimal dose regimen should be further screened. Lastly, the immune modulating effect of biomaterials involved in these nanoplatforms should be further explored. Sometimes, the nanomaterial could demonstrate immune stimulatory or suppressive effect, for example, nanomaterials composed of Al_2_O_3_ or polysaccharides were reported to have adjuvant effect 109-111.

Despite the excellent therapeutic effect of combined PTT and immunotherapy on animal studies, clinical translation still faces many challenges. The specific mechanisms of immune responses induced by PTT are complicated, and the intensity and controllability of immune responses during combination therapy are also continually to be studied. Moreover, the potential risks of PTT to patients caused by toxicity of nanomaterials, hyperthermia or laser power density should not be ignored. For example, the high temperature or prolonged irradiation time aiming to obtain stronger thermal ablation might result in unexpected damage to noncancerous tissue nearby. The safe power density limit is ~0.33 W cm^-2^ for the 808 nm NIR laser and ~0.726 W cm^-2^ for the 980 nm NIR laser as indicated by American National Standard for the Safe Use of Lasers, but there was higher power density being used in the above mentioned studies 112. Also, the safety for immunotherapy should be explored further. The commonly seen immune related side effects (irAE) including various inflammation, neurological toxicity, anaphylaxis and cytokine release syndrome should be also be noted 113. Currently, all of these studies are in the laboratory stage, and the feasibility and safety of these innovatively designed nanoplatforms need to be further studied before they are used in the clinic. Even so, the combination of PTT and immunotherapy still shows great potential to become an important complement for tumor treatments.

## Figures and Tables

**Table 1:** Photothermal materials combined with immune modulators for photothermal-immunotherapy

Photothermal agent	Type	Nanocarrier	Immunotherapy	Immunotherapy agent	Cancer model	Therapeutic outcome	References
pD	organic	MPDA NPs	Immune adjuvant	R837	B16F10	Significantly suppressed primary tumor growth and notably prolonged the survival time of mice	58
PANI	organic	GCS	Immune adjuvant	R848	CT26	Almost completely restrained the primary tumor and effectively suppressed the tumor recurrence and metastasis	61
pD	organic	Al_2_O_3_ NPs	Immune adjuvant	Al_2_O_3_, CpG ODNs	B16F10	Significantly inhibited the primary tumor and prolonged the survival time of mice	67
IR7	organic	Liposomes	Immune adjuvant	CpG ODNs	CT26	Effectively eradicated primary tumors in mice and inhibited tumor metastasis.	69
ICG	organic	Liposomes	Immune adjuvant	Poly I:C	CT26, B16F10	Almost completely restrained the primary tumor growth and prevented metastasis of cancer	72
ICG	organic	PLEL hydrogel	Immune adjuvant	R848, CpG ODNs	4T1	Inhibited the residual tumor growth after surgery, prevented recurrence and metastasis	76
ICG	organic	Fe_3_O_4_ NPs	Immune adjuvant	R837	4T1	Inhibited tumor growth, metastasis and recurrence	14
BPQDs	inorganic	Hydrogel	Immune adjuvant, Immune checkpoint inhibition	LPS, GM-CSF, PD-1 antibody	4T1-luc, B16F10-luc	Inhibited the recurrence and metastasis of tumors and prolonged the survival rate	23
ICG	organic	OVA	Exogenous tumor antigen	OVA	B16	Almost totally inhibited the primary tumor growth and prevented occurrence of tumor	50
BPQDs	inorganic	Exosomes	Exogenous tumor antigen	Exosomes	LLC	Significantly delayed tumor occurrence and almost totally inhibited the primary tumor growth	52
ICG	organic	Eukaryotic-pr-okaryotic vesicle, PLGA	Exogenous tumor antigen, adjuvant	Melanoma cytomembrane vesicles, attenuated Salmonella outer membrane vesicles	B16	Almost totally inhibited primary tumor and efficiently suppressed tumorgenesis	78
MnFe_2_O_4_	inorganic	MnFe_2_O_4_ NPs	Exogenous tumor antigen, adjuvant	OVA, R837	4T1	Effectively restrained primary tumor growth and prevented lung metastases, resulting in a prolonged survival time and improved survival rate	114
Au@Pt NPs	inorganic	Au@Pt NPs	Immune checkpoint inhibition	PD-L1 antibody	4T1	Effectively eliminated primary tumors, inhibited the growth of distal tumors and alleviated tumor metastasis	31
BPQD	inorganic	Nanovesicle	Immune checkpoint inhibition	PD-1 antibody	4T1	Significantly inhibited primary and secondary tumor growth	83
HAuNS	inorganic	PLGA	Immune checkpoint inhibition	PD-1 antibody	CT26, 4T1	Efficiently eliminated most primary tumors, significantly inhibited the growth of the distant tumors, and induced the longest survival time	84
PBNP	inorganic	PBNP	Immune checkpoint inhibition	CTLA-4 antibody	Neuro2a	Decreased tumor growth rates and exhibited protection against tumor re-challenge	87
Melanin nanoparticles	organic	Melanin NPs	IDO inhibition	INCB24360	4T1	Strongly inhibited both primary and abscopal 4T1 tumors	90
Fe_3_O_4_ NPs	inorganic	PLGA	Immune checkpoint inhibition, immune adjuvant	R837, PD-1 antibody	4T1	Significantly inhibited both the primary and distant tumors and effectively prevented the lungs/liver metastasis	94
ICG	organic	Covalent organic frameworks	Exogenous tumor antigen, immune checkpoint inhibition	OVA, PD-L1 antibody	CT26	Effectively eliminated primary tumor and inhibited the metastasis of cancer cells	115
Bi_2_Se_3_ nanocage	inorganic	Bi_2_Se_3_ nanocage	Immune checkpoint inhibition, immune adjuvant	R848, PD-L1 antibody	4T1	Totally inhibited both the primary tumor and distant tumor and established long-term immune memory	95
ICG	organic	Liposome	Immune checkpoint inhibition	PD-1 antibody, TIM-3 antibody	MC38, CT26	Cleared the primary tumor and inhibited the growth of the distant tumor	93
Au_40C_-DOPC	inorganic	PLGA	Immune checkpoint inhibition, immune adjuvant	R837, PD-L1 antibody	4T1	Significantly suppressed both the primary tumor and distant tumor	116
IR1061	organic	PAAV-SNO	IDO inhibition	IDO inhibitor	4T1	Significantly inhibited the primary tumor growth with 4/6 mice became tumor free and suppressed breast lung metastasis	97
ICG	organic	PLGA	CAR-T therapy	CAR-T cells	WM115	Significantly inhibited the primary tumor growth with 2 out of 6 mice being completely cured	100
GNR	inorganic	GNR	Immune checkpoint inhibition, STING	PD-1 antibody, STING	K7	Strongly inhibited the primary tumors and significantly retarded distant tumors	103
Au NPs	inorganic	Au NPs and TNF-α	Cytokine therapy	TNF-α	4T1	Significantly inhibited the primary 4T1 tumors	104
CuS	inorganic	MSN	Cytokine therapy	IL-12	B16F10	Effectively inhibited both primary and distant tumors	105

## Abbreviations

APCs: antigen presenting cells
AuNSs: gold nanostars
BPQDs: black phosphorus quantum dots
CAR-T: chimeric antigen receptor T cells
CD: carbon nanodots
CRT: calreticulin
CpG ODNs: cytosine-phosphate-guanine oligodeoxynucleotides
CTLs: cytotoxic T lymphocyte
CTLA-4: cytotoxic T lymphocyte-associated antigen 4
CXCL10: CXC chemokine ligand-10
DAMPs: damage-associated molecular patterns
DCs: dendritic cells
Dox: doxorubicin
DLNs: draining lymph nodes
ER: endoplasmic reticulum
GCS: glycol-chitosan
GM-CSF: granulocyte-macrophage colony stimulating factor
GNPs: gold nanoparticles
GNRs: gold nanorods
HMGB1: high mobility group box 1
HSP: heat shock proteins
IDO: indoleamine 2,3-dioxygenase
IFN-γ: interferon-γ
ICB: immune checkpoint blockade
ICD: immunogenic cell death
ICG: indocyanine green
IRF3: interferon regulatory factor 3
ITM: immunosuppressive tumor microenvironment
LPS: lipopolysaccharide
IgG: immunoglobulin G
IL-10: interleukin-10
IL-12: interleukin-12
ITM: immunosuppressive tumor microenvironment
LNs: lymph nodes
MDSC: myeloid-derived suppressor cell
MSN: mesoporous silica nanoparticles
MHC: major histocompatibility complex
MPDA NPs: mesoporous polydopamine nanoparticles
MPLA: monophosphoryl lipid A
NIR: near-infrared
OVA: ovalbumin
PAMPs: pathogen associated molecular patterns
PANI: polyaniline
PBNP: prussian blue nanoparticle
pD: polydopamine
PD-1/PD-L1: programmed cell death 1/programmed death ligand 1
PDT: photodynamic therapy
poly I:C: polyinosinic:polycytidylic acid
TRL: thermo-responsive liposomes
PTT: photothermal therapy
RM: erythrocyte membrane
TAAs: tumor-associated antigens
TDLNs: tumor draining lymph nodes
TGF-β: transforming growth factor β
TIM-3: T cell immunoglobulin and mucin domain containing protein 3
TME: tumor microenvironment
TLR: Toll-like receptor
Tregs: regulatory T cells
STING: stimulator of interferon gene
SWCNT: single-walled carbon nanotubes
